# The Tumor Targeted Superantigen ABR-217620 Selectively Engages TRBV7-9 and Exploits TCR-pMHC Affinity Mimicry in Mediating T Cell Cytotoxicity

**DOI:** 10.1371/journal.pone.0079082

**Published:** 2013-10-23

**Authors:** Gunnar Hedlund, Helena Eriksson, Anette Sundstedt, Göran Forsberg, Bent K. Jakobsen, Nicholas Pumphrey, Karin Rödström, Karin Lindkvist-Petersson, Per Björk

**Affiliations:** 1 Active Biotech AB, Lund, Sweden; 2 Immunocore Ltd., Oxon, United Kingdom; 3 Adaptimmune Ltd., Oxon, United Kingdom; 4 Experimental Medical Science, Lund University, Lund, Sweden; University of Pittsburgh, United States of America

## Abstract

The T lymphocytes are the most important effector cells in immunotherapy of cancer. The conceptual objective for developing the tumor targeted superantigen (TTS) ABR-217620 (naptumomab estafenatox, 5T4Fab-SEA/E-120), now in phase 3 studies for advanced renal cell cancer, was to selectively coat tumor cells with cytotoxic T lymphocytes (CTL) target structures functionally similar to natural CTL pMHC target molecules. Here we present data showing that the molecular basis for the anti-tumor activity by ABR-217620 resides in the distinct interaction between the T cell receptor β variable (TRBV) 7-9 and the engineered superantigen (Sag) SEA/E-120 in the fusion protein bound to the 5T4 antigen on tumor cells. Multimeric but not monomeric ABR-217620 selectively stains TRBV7-9 expressing T lymphocytes from human peripheral blood similar to antigen specific staining of T cells with pMHC tetramers. SEA/E-120 selectively activates TRBV7-9 expressing T lymphocytes resulting in expansion of the subset. ABR-217620 selectively triggers TRBV7-9 expressing cytotoxic T lymphocytes to kill 5T4 positive tumor cells. Furthermore, ABR-217620 activates TRBV7-9 expressing T cell line cells in the presence of cell- and bead-bound 5T4 tumor antigen. Surface plasmon resonance analysis revealed that ABR-217620 binds to 5T4 with high affinity, to TRBV7-9 with low affinity and to MHC class II with very low affinity. The T lymphocyte engagement by ABR-217620 is constituted by displaying high affinity binding to the tumor cells (K_D_ approximately 1 nM) and with the mimicry of natural productive immune TCR-pMHC contact using affinities of around 1 µM. This difference in kinetics between the two components of the ABR-217620 fusion protein will bias the binding towards the 5T4 target antigen, efficiently activating T-cells via SEA/E-120 only when presented by the tumor cells.

## Introduction

T lymphocytes have been attributed the potential to extinguish large malignant tumors and therefore activation and regulation of these cells are corner stones in developing immune therapy of cancer. To reject established tumors, selective T cells have to multiply and invade the tumor in sufficient numbers and with an anti-tumor effector-function profile. There is now substantial evidence showing that the immune system, in particular the CD8^+^ T cell subset, has the ability to recognize and eliminate established tumors [1–6]. Effective anti-tumor T cell responses require high numbers of selective T cells similar to productive responses to acute virus infections. To achieve such strong T cell responses and to reset the often immune suppressive tumor microenvironment, distinct vaccination schedules and adoptive T cell therapies have been developed and tested with success [1–6]. 

Despite the complex interactions realized in the immunological synapses between cytotoxic T lymphocytes (CTLs) and target cells [7,8], the selective activation of anti-tumor T cells reside in the balanced affinity between the T cell receptor (TCR) and the molecular target complex of tumor associated antigen (TAA) peptides bound to the MHC products (pMHC) on the targeted tumor cells. The TCRs are cell membrane integrated proteins that bind to cell membrane pMHCs and, in contrast to antibodies (Abs), do not usually act as monomeric binders of antigen in solution. The affinities of TCRs are normally low as compared to maturated Abs [9] and the TCRs rely on multimeric interactions between the effector T cell and the target cell membranes. This is illustrated by showing selective and strong staining of T cells with fluorochrome-conjugated multimeric pMHC protein complexes and no staining with pMHC monomers [10,11]. An optimal T cell activation signal can be achieved with TCR-pMHC interactions representing fast on-rate and a K_D_ in the lower micromolar range with as few as 3-25 pMHC per target cell [9]. Sykulev et al. showed that TCR-pMHC affinities around 1 µM were productive with low pMHC density cell expression in a model for TCR dependent CTL activation [12]. 

We have previously constructed the tools for and applied a new strategy to achieve strong selective T cell activation towards established human tumors, the tumor-targeted superantigens (TTSs) [13–15]. A tumor specific superantigen (Sag) can be created by fusing the Sag to the Fab moiety of a tumor reactive monoclonal antibody (mAb) (Schematic depictions of different TTSs in Figure S1). TTSs activate and induce multiplication of a distinct subset of T cells and direct them to the tumor cells by means of the fusion proteins between bacterial Sags and Fab-fragments of tumor-reactive mAbs. Sags are bacterial and viral proteins that share the ability to activate a large number of T lymphocytes. Bacterial Sags bind to MHC class II molecules proteins and subsequently interact with T cells expressing particular TCR Vβ chains [16–18]. Sags are efficient inducers of inflammatory cytokine production and CTL-mediated cytotoxicity [19–21]. To target the Sag-induced T cell activity against tumor cells, Fab regions of tumor-reactive monoclonal antibodies have been genetically fused with wild-type Sags or engineered variants thereof, e.g. the staphylococcal enterotoxin (SE) A (Fab-SEA) [14,22]. The therapeutic efficacy is ascribed to the dual mechanism of tumor cell killing: direct lysis of tumor cells expressing the antigen recognized by the antibody moiety of the fusion protein and secretion of cytokines eliminating antigen-negative tumor cell variants (Schematic illustration of the proposed TTS basic mechanism of action in Figure S2). The effectiveness of this therapy has been demonstrated both in syngeneic and xenogeneic tumor models in mice [14,22,23]. TTSs are in clinical development and have shown promising results, for example in patients with advanced non-small cell lung cancer (NSCLC) and renal cell carcinoma (RCC) using ABR-214936 (aka anatumomab mafenatox) [24,25]. Pharmacological proof-of-concept in man was obtained by a series of observations in subsequent phase 1 clinical studies of ABR-217620 (aka naptumomab estafenatox and 5T4Fab-SEA/E-120), including dose-dependent induction of IL-2 and IFN-γ, selective expansion of T cells as well as tumor infiltration of T lymphocytes in tumor biopsies from ABR-217620 treated patients [26,27].

ABR-217620 is an engineered TTS with a distinct pharmacological profile and consists of a recombinant fusion protein developed from ABR-214936. ABR-217620 consists of a mutated variant of the Sag See containing distinct epitopes of SEA (SEA/E-120) [28] linked to a Fab-moiety of a monoclonal antibody recognizing the tumor-associated oncofetal trophoblast glycoprotein antigen 5T4 [29]. This therapeutic fusion protein was constructed to target tumor cells with high affinity and, when bound, to recruit and activate T cells through low affinity interactions with the TCR. SEA/E-120 has low MHC class II binding affinity to prevent cytokine-induced systemic toxicity and structural modifications to reduce immunogenicity. Here we show that ABR-217620 selectively binds to and activates T cells expressing T cell receptor β variable (TRBV) 7-9 (according to IMGT nomenclature; TCRVβ6.4 according to Arden et al. [30] nomenclature) and induces CTL activation using T cell/tumor cell affinity mimicry of natural productive T cell responses.

## Materials and Methods

### Cells and reagents

The human kidney cancer cell line Caki-2 and the human T cell line J.RT3-T3.5 [31,32], a mutated form of the Jurkat cell line that has a defect in the expression of endogenous TCRVβ-chain, were obtained from the ATCC (American Type Culture Collection, Rockville, MD). Human peripheral blood mononuclear cells (PBMC) were isolated from healthy donors. Blood was drawn from healthy volunteers at occupational health department at Active Biotech AB. After recommendation from the local Ethics Committee in Lund, Sweden, no formal ethical approval was needed. The blood donation was performed under consent and according to waiver LU322-03 executed by Lund University Research Ethics Committee and according to Swedish law 2003:460. Briefly, blood was collected by venipuncture into Vacutainer CPT tubes (BD, Stockholm, Sweden) containing sodium citrate and the PBMCs were isolated as described by the manufacturer. The SEA- and SEA/E-120-reactive T cell lines were established from human peripheral blood lymphocytes as described previously [33]. All cells were cultured in R10 medium (RPMI 1640 medium with ultraglutamine (Cambrex Bio Science, Walkersville, USA), supplemented with 10% FCS (Sigma-Aldrich), 1 mM sodium-pyruvate (Cambrex Bio Science), 0.1 mg/ml Gentamicin sulfate (Cambrex Bio Science)) under standard conditions. 

Mouse monoclonal anti-human 5T4 (Mab5T4-H8) was produced at Active Biotech and the mouse monoclonal anti-human CD2 purchased from Abcam, UK. Fluorochrome-conjugated anti-human CD3, CD4, CD8 and HLA-DR were obtained from BD Biosciences (San Diego, CA). Recombinant human CD28 and EpCAM (identical to the C215 antigen [34]) fused with a human IgG_1_Fc tag, were purchased from R&D Systems (Minneapolis, MN). Recombinant ABR-217620, 5T4Fab(V18)-SEA, C215Fab-SEA/E-120, C215Fab-SEE, SEA, SEE and SEA/E-120 were expressed as secreted proteins in *E. coli* as described elsewhere [28,35,36]. ABR-217620 was biotinylated using EZ-Link Sulfo-NHS-LC-Biotin (Pierce, Rockford, IL) according to manufacturer’s instructions. Recombinant human 5T4-IgG_1_Fc (5T4Fc) was obtained from Peter L Stern (Paterson Institute for Cancer Research, University of Manchester, Manchester, UK). The soluble TCR reagent TRBV7-9 was produced as a disulfide-linked αβ-heterodimer as described by Boulter et al. [37]. Recombinant soluble human MHC class II (HLA-DR1) α and β extracellular domains, were produced separately as inclusion bodies in *E. coli*. Purified domains were allowed to assemble *in vitro* in the presence of with *Hemophilus* influenza agglutinin peptide (sequence PKYVKQNTLKLAT) as described by Frayser et al. [38] with minor modifications [39].

### Cloning of the TRBV7-9 Cβ1 (TRBC1) TCR chain

cDNA encoding the full-length rearranged TRBV7-9 Cβ1 (TRBC1) TCR chain was cloned from a SEA/E-120 reactive T cell line. Briefly, total RNA was extracted from the SEA/E-120 reactive T cell line using Qiagen RNeasy Mini Kit (QIAGEN, Hilden, Germany). First-strand cDNA was obtained by using Transcriptor First Strand cDNA Synthesis Kit (Roche Diagnostics GmbH, Mannheim, Germany) and oligo(dT) primer according to manufacturer’s instructions. Subsequently, the cDNA was amplified by using the AccuPrime^TM^ *Pfx* SuperMix (Invitrogen, Carlsbad, CA) and the forward 5’-TTGGCGCGCATGGGCACCAGCCTCC-3’ and reverse 5’-TCCCCCGGGATGACGGGTTAGAAGCTCCTAAC-3’ primers for 35 cycles (denaturation 95 °C for 15 s, annealing 55 °C for 30 s, extension 68 °C for 60 s). The introduced BssH II site in the forward primer and the Sma I site in the reverse primer are underlined. The PCR product, consisting of 950 bp, was then ligated into the pIREShyg3 mammalian expression vector (Clontech Laboratories Inc., Mountain View, CA), and sequenced.

### Establishment of a TRBV7-9^+^NFκB-Luc^+^ J.RT3-T3.5 cell line

The vector encoding the TRBV7-9 Cβ1 (TRBC1) TCR chain and an NFκB-luciferase reporter vector (Clontech Laboratories Inc.) were sequentially transfected into the J.RT3-T3.5 cells. Briefly, 25 µg of the pIREShyg3-vector containing full-length TRBV7-9 Cβ1 TCR chain was transfected into J.RT3-T3.5 cells by electroporation using a Bio-Rad Gene Pulser II (Bio-Rad, Hercules, CA) with the voltage set at 260 V and capacitance set at 960 µF. The cells were incubated for 10 min at room temperature, resuspended in fresh R10 medium and then cultured at 37 °C. After 48 hours, cells were distributed in 96-well plates at a cell density of 5000 cells/well and cultured for 3 weeks in the presence of hygromycin B (0.4 mg/ml; Invitrogen). Individual clones growing in the selection medium were expanded to mass cultures and assayed for TRBV7-9-expression by flow cytometry using the [ABR-217620-biotin/SA-PE]-complex as described below.

NFκB-luciferace reporter vector (25µg) was then cotransfected with 10 µg of a neomycin resistance vector (pcDNA3; Invitrogen) into a TRBV7-9 expressing J.RT3-T3.5 cell clone by electroporation as described above. Clones growing in the presence of 0.4 mg/ml hygromycin B and 1 mg/ml G418 Sulfate (Calbiochem, La Jolla, CA) were expanded to mass cultures and screened for high luciferase activity, as described below, upon nonspecific stimulation with phorbol myristate acetate (PMA; Sigma, St. Louis, MO) and ionomycin (Sigma). 

### Luciferase Reporter Assay

NFκB activation in the TRBV7-9^+^NFκB-Luc^+^ J.RT3-T3.5 cells was measured by the production of luciferase upon stimulation by using the PerkinElmer Steadylite plus High Sensitivity Luminescence Reporter Gene Assay System (Boston, MA) according to manufacturer’s instructions. Activity was expressed in relative luminescence units (RLUs). 

### Activation of TRBV7-9^+^NFκB-Luc^+^ J.RT3-T3.5 cells by ABR-217620 presented upon Caki -2 cells

10^5^ TRBV7-9^+^NFκB-Luc^+^ J.RT3-T3.5 cells were co-cultured with 5x10^4^ Caki-2 cells and different concentrations of ABR-217620 or SEA/E-120 for 6 h. The NFκB activity was measured as described above.

### Activation of TRBV7-9^+^NFκB-Luc^+^ J.RT3-T3.5 cells by ABR-217620 presented upon on latexbeads

Latexbeads (Sulfate latex 4% w/v 5 µM, Invitrogen, USA) were coated with 5T4Fc and mouse monoclonal anti-human CD2 according to manufacturer’s instructions.

The TRBV7-9^+^NFκB-Luc^+^ J.RT3-T3.5 cell clone were preincubated with 100 µg/ml Mab5T4 in medium on ice for 30 minutes and washed. 10^5^ of these J.RT3-T3.5 cells were then co-cultured with 2x10^5^ coated latexbeads and different concentrations of ABR-217620 for 4 h. The NFκB activity was measured as described above.

### FACS analysis and sorting

FACS analyses were done on using a FACSort flow cytometer (BD Biosciences) or a FACSCanto II flow cytometer (BD Biosciences). The TRBV7-9 expression on T cells was identified by using a flow cytometric method based on staining with a fluorochrome-conjugated multimeric ABR-217620. Briefly, Biotin-conjugated ABR-217620 and Streptavidin-PE (BD Bioscience, San Jose, CA) were mixed and allowed to form complexes to a final molar ratio of approximately 6:1 (for selection of molar ratio see Figure S3). Cells were then resuspended in the fluorochrome-conjugated multimeric ABR-217620 solution and stained with appropriate FITC-conjugated mAb according to standard procedures. Single and viable cells that bound the fluorochrome-conjugated multimeric ABR-217620 were selected for sorting based on the laser scatter profile and fluorescence intensity in red fluorescence. Cell sorting was performed on a FACSort flow cytometer or a BD FACSAria™ flow cytometer (BD Biosciences).

### Analysis of TCRVβ usage

Total RNA was prepared from PBMC or activated T-cell lines (approximately 7x10^6^ cells/sample) using RNAqueous -4PCR (Ambion, Huntingdon, UK). cDNA was synthesized from 1 µg total RNA/sample using 1st strand cDNA synthesis kit for RT-PCR (AMV; Roche, Basel, Switzerland). The TCR-Vβ profiling was performed by TcLand SA (Nantes, France). The TcLandscape® technology provides both a global and precise picture of T cell mobilization by combining a quantitative and qualitative assessment of TCR gene usage [40]. The quantitative PCR assessment was done for 26 TCR-Vβ chains / chain families. Experimental results are provided as amounts of transcripts of Vβ chains / chain families (in cDNA copies) depicted as quantitative Vβ/HPRT ratios.

### T cell cytotoxicity assay

Cytotoxicity was measured against Caki-2 cells with or without ABR-217620 using a standard 4 hr ^51^Cr-release assay [14]. The percent specific cytotoxicity was calculated as 100 x [(cpm experimental release – cpm background release)/(cpm total release – background release)]. SEA-reactive human T cell lines were used as effector cells at an effector to target ratio of 45:1. ^51^Cr-labelled Caki-2 target cells were used at 2.5 x 10^6^ cells per well in V-bottomed 96 well microtiter plates.

### Surface plasmon resonance (SPR) analysis

SPR analyses were carried out at 25 °C using the Biacore 3000 system from GE Healthcare. CM5 sensor chips, amine coupling kit and buffers were obtained from GE Healthcare. Buffer of reagents was changed to 10 mM Hepes, 0.15 M NaCl, pH 7.4, containing 0.005% (v/v) Surfactant-P-20 (HBS-P) on micro spin columns prior to binding experiments. Evaluation of binding data was made using BIAevaluation software version 3.2 (GE Healthcare) and curve fitting in GraphPad Prism 4 (GraphPad Software, Inc., La Jolla, CA).

Binding of Fab moiety to 5T4 antigen: 5T4Fc was immobilized on a CM5 chip by amine coupling at a concentration of 55 µg/ml in 10 mM Na acetate buffer, pH 4.5, to the desired level. Serially diluted ABR-217620 (concentration range 1.56-50 nM) was injected for 3 min at 20 µl/min with C215Fab-SEA/E-120 as negative control. Regeneration of the surface was made by a 15 µl pulse of 10 mM glycine-HCl, pH 1.5, during 30 s.

Soluble TCR interaction with Sag: 5T4Fc, EpCAMFc and CD28Fc were immobilized at low densities (< 1 kRU) by amine coupling. In a first step, Sags were captured via their 5T4, C215 or CD28 Fab fusion partner followed by a second step where TRBV7-9 in the concentration range from 62.5 to 1,000 nM were allowed to interact with the SEA, SEE or SEA/E-120 moiety.

Interaction of MHC class II/HA peptide with Sag: 50 nM SEA, SEE and SEA/E-120 were first captured on either immobilized 5T4Fc (ABR-217620 and 5T4Fab-SEA) or EpCAMFc (C215Fab-SEE). pHLA-DR1 was then allowed to interact with the captured Sag at concentrations ranging from 0.625 to 5 µM for kinetic analysis. Dilution of pHLA-DR1 was made in HBS-P containing 10 µM Zn^2+^.

### Analysis of TCRVβ binding pattern

To perform a detailed analyses on which amino acids that contribute to the narrowing of the TCVβ profile of SEA/E-120, contacting residues between SEB and TCRVβ were calculated using the program CONTACT in the CCP4 suite of program [41], using the SEB-TCRVβ structure (PDB ID:1sbb) [42]. The residue-areas from SEB that contact TCRVβ in the structure, were mapped onto SEA structure (PDB ID:1lo5) [43] and are likely to be involved in the interaction with TCRVβ for SEA as well as for SEA/E-120. The SEA, SEE, and SEA/E-120 sequences were aligned using ClustalW and the model was generated using PyMOL (The PyMOL Molecular Graphics System, DeLano WL, Scientific LLC, 2008).

## Results

### Multimeric ABR-217620 selectively binds to TRBV7-9 expressing T lymphocytes from human peripheral blood

**Figure 1:**
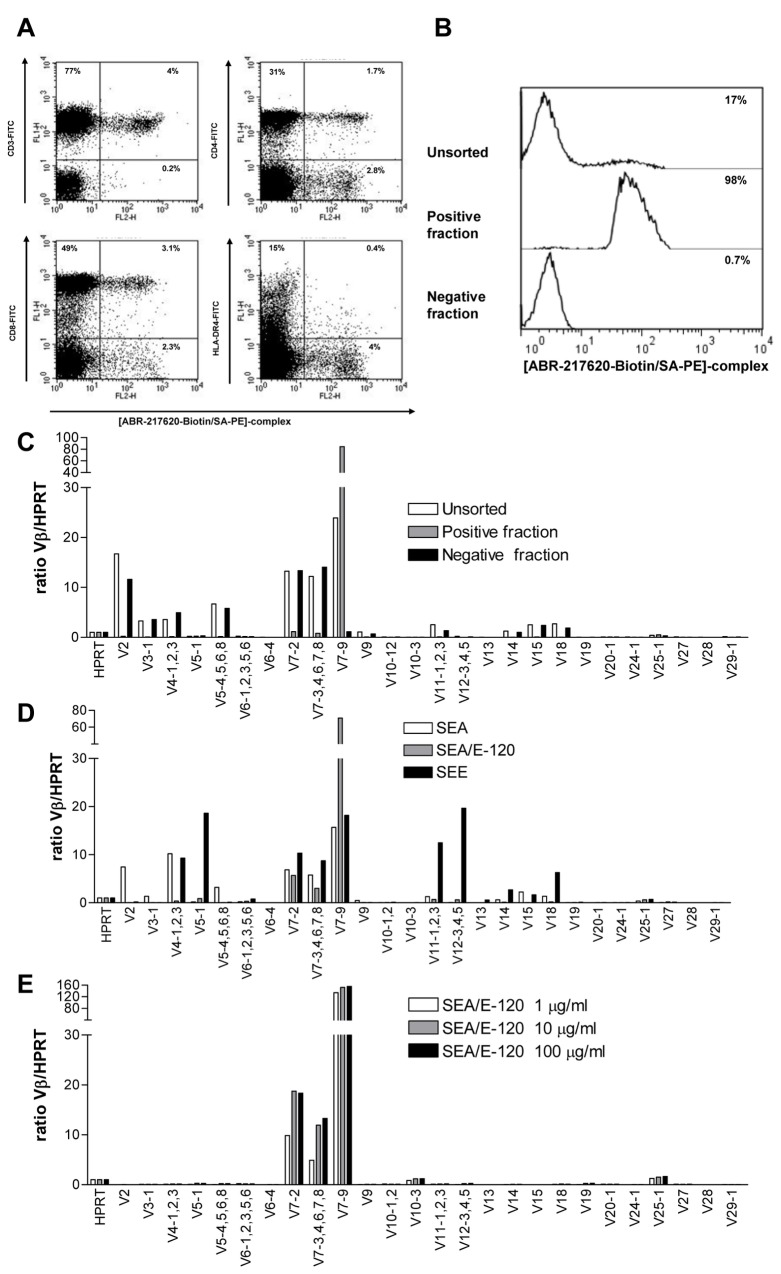
Flow cytometry and TCR-mRNA (IMGT TRB variants) analysis. A) Flow cytometry analysis of [ABR-217620-Biotin/SA-PE]-complex binding to human PBMC from a healthy donor also stained for CD3, CD4, CD8 and HLA-DR. B) Re-analysis of flow cytometry sorted human PBMC from a healthy donor and C) TCR-mRNA analysis (IMGT TRB-variants) of unsorted and sorted cells binding or not binding the [ABR-217620-biotin/SA-PE]-complex. D) TCR-mRNA analysis (IMGT TRB-variants) of T cells from *in* *vitro* cultures activated with SEA, SEA/E-120 or SEE. E) TCR-mRNA analysis (IMGT TRB-variants) of T cells from *in* *vitro* cultures activated with different concentrations of SEA/E-120.

ABR-217620 containing the engineered Sag SEA/E-120 activates human T cells *in vitro* and *in vivo* despite the diminished MHC class II binding [26,29]. ABR-217620 showed no binding to T cells using conventional FACS staining procedures. In an attempt to define potential low affinity ABR-217620 binding T cells, we made fluorochrome-conjugated multimeric ABR-217620 in analogy with the pMHC tetramer approach [11] and stained fresh blood lymphocytes from healthy control individuals. Multimeric ABR-217620 bound to approximately 5% of the peripheral blood lymphocytes representing only CD3^+^ T cells and including both the CD4^+^ and the CD8^+^ subsets (Figure 1A). Most Sags (including SEA and SEE) have distinct TCRVβ binding selectivity and quantitative RT-PCR-analysis of blood lymphocytes from cancer patients treated with ABR-217620 indicated that preferentially TRBV7-9 expressing T cells were activated and expanded [26]. T cells from a healthy control individual were stained with multimeric ABR-217620, separated on a FACS cell sorter (Figure 1B) and analyzed for TCRVβ-expression using quantitative RT-PCR. ABR-217620 binding T lymphocytes were highly enriched for expression of TRBV7-9 while the non-binders were essentially negative for this variant of TCR (Figure 1C). Thus, multimeric ABR-217620 selectively binds to T cells expressing TRBV7-9.

### ABR-217620 selectively activates TRBV7-9 expressing T lymphocytes

We recently reported that ABR-217620 activates human T lymphocytes *in vitro* [29], resulting in cytokine production, proliferation, differentiation and ABR-217620 directed CTL cytotoxicity. To analyze the potential distinct TCRVβ activation selectivity of the SEA/SEE hybrid protein SEA/E-120 the repertoires of TCRVβ families in SEA and SEE stimulated T cell lines were compared to the TCRVβ profile in a T cell line activated by SEA/E-120 from the same healthy control blood donor. The results from quantitative RT-PCR analysis of TCRVβ mRNA showed that the ranges of TCRVβ families activated by SEA and SEE were similar to previous data [44]. However, the reactivity to SEA/E-120 was restricted to members of the TRBV7 family, in particular TRBV7-9 (Figure 1D). T cells expressing TRBV7-2 and TRBV7-3,-4,-6,-7-8 (the primers detected several TRBV7 family members) were also expanded, although to a much lower degree. In order to address if increasing the Sag concentration would allow expansion of T cells expressing TCRVβ with lower affinity for SEA/E-120, T cell lines were established using three different doses of Sag. The human T cells were stimulated repeatedly with SEA/E-120 at 1, 10 or 100 µg/ml and analyzed for TCRVβ expression after six weeks of culture. The results demonstrated a strong dominance of T cells expressing TRBV7-9 at all investigated doses (Figure 1E), arguing against other TCRVβs being activated at higher concentrations of SEA/E-120. These results clearly show that the TCRVβ specificity of the engineered Sag part of ABR-217620, SEA/E-120, is restricted to members of the TRBV7 family, in particular TRBV7-9. We recently reported that ABR-217620 activates T lymphocytes *in vivo* showing T cell cytokine production and selective expansion of blood T cell TRBV7-9 mRNA in cancer patients after treatment with ABR-217620 [26]. Taken together, this demonstrates that ABR-217620 selectively binds and activates T cells expressing TRBV7-9 to proliferation and expansion.

**Figure 2:**
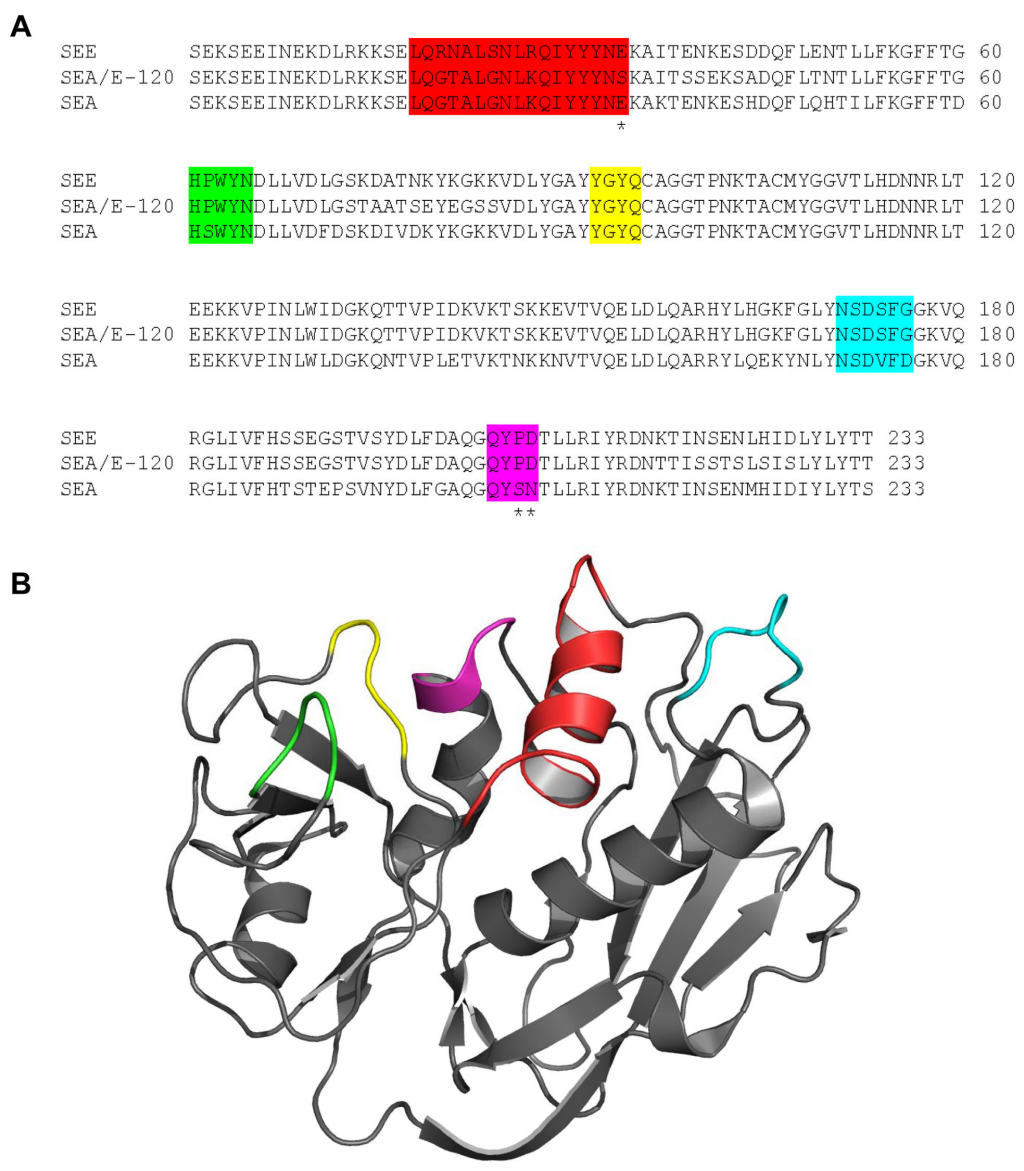
Sequences and structure of Sags show putative TCR binding regions on SEA, SEE and SEA/E-120. A) Sequence alignment of the SEA/E-120, SEA and SEE. Residues in the presumed TCR binding site are colored. Residues believed to be of particular importance for TCRVβ binding are marked with asterisks. B) Putative TCR binding residues are mapped onto the SEA structure (PDB ID: 1lo5) and marked in accordance with the coloring in A.

With the SEB-TCRVβ structure as a model, a detailed sequence analysis of SEA/E-120 revealed five parts of the protein in close contact with TCRVβ (Figure 2). One of these parts, Tyr91 to Glu94 (given in accordance with SEA/E-120 nomenclature, colored green in Figure 2), is fully conserved between SEA, SEE, and SEA/E-120 and hence would not contribute to the narrowing of the TCRVβ profile for SEA/E-120. In the four remaining parts, there is one or more than one amino acids in each section of SEA/E-120 that either is the same as in SEA, SEE or different from both of them. In the first region (colored red in Figure 2), Gln19 to Ser34, the sequence of SEA/E-120 is almost identical to SEA but interestingly there is a serine (Ser34) that differ both from SEA and SEE, which have glutamates instead. A serine is smaller in size and has different polarity and hence could very well be one of the more important amino acids contributing to the narrowing of the TCRVβ profile for SEA/E-120. The three remaining parts, His61 to Asn65 (yellow), Asn171 to Gly176 (cyan), and Glu204 to Asp206 (magenta), have five amino acids in total that are identical to SEE instead of SEA. Since SEA has a broader TCRVβ specificity than SEE 45, consequently the TCRVβ specificity will be narrowed by having SEE-like amino acids in the SEA/E-120 sequence of the TCR binding regions. Moreover, Pro206 and Asp207 (magenta) in SEE are identical in SEA/E-120 but correspond to Ser206 and Asn207 in SEA. These residues are located at the outer rim of the TCR-binding site and have been shown to determine the TRBV28 and TRBV25-1 specificities [46]. Since SEA/E-120 is identical to SEE at these points, a minor activation of TRBV25-1 was expected, as seen in Figure 1E. 

### ABR-217620 activates TRBV7-9 expressing cytotoxic T lymphocytes to kill 5T4 positive tumor cells

**Figure 3:**
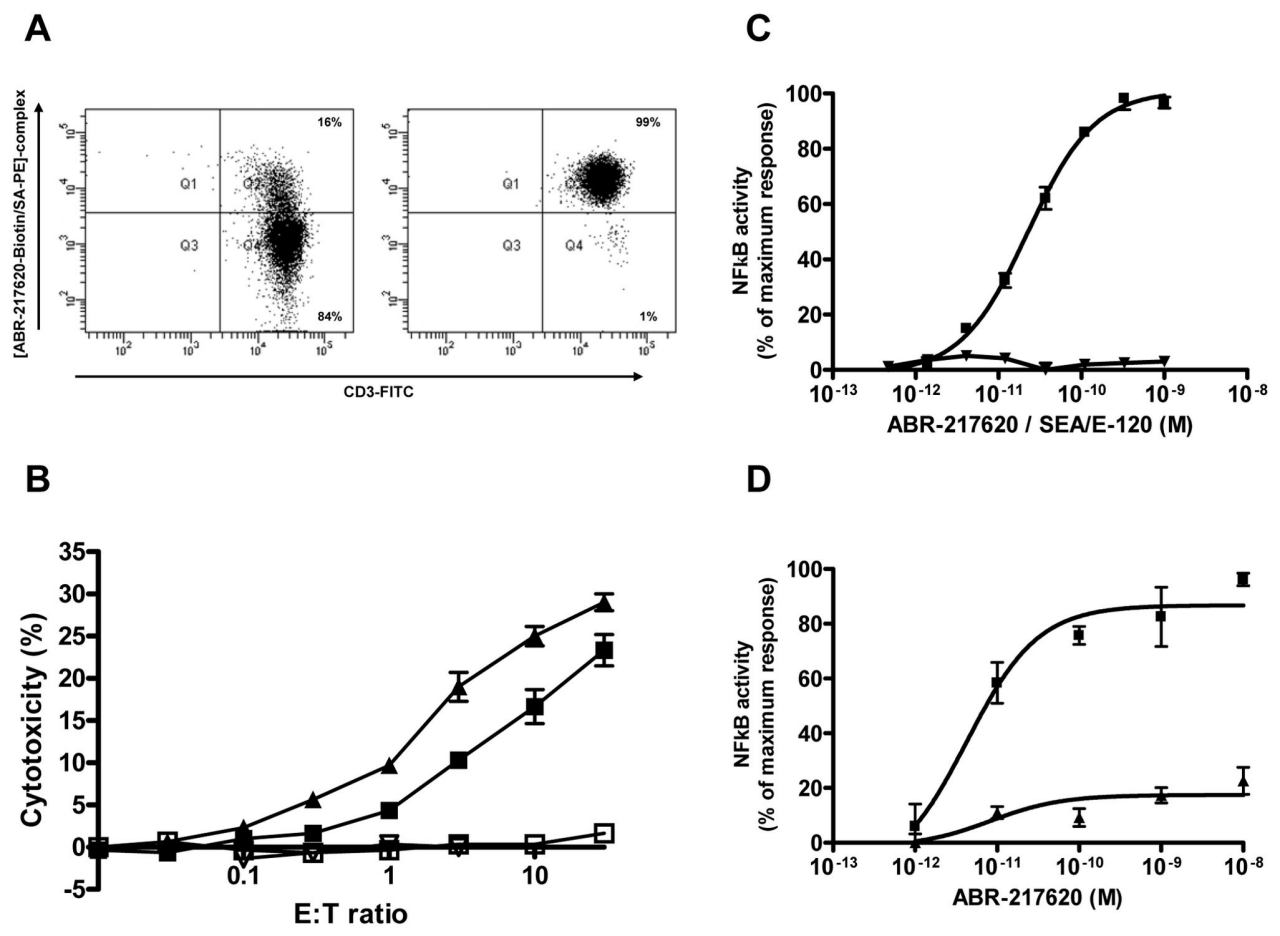
Flow cytometry, cytotoxicity (SADCC) and NFκB-luciferace reporter gene analysis. A) Analysis of flow cytometry sorted SEA activated T cells and B) cytotoxicity (SADCC) measured against Caki-2 cells with (filled) or without (open) ABR-217620 using a standard 4 hr ^51^Cr-release assay of unsorted (squares) and sorted (triangles) cells binding the [ABR-217620-biotin/SA-PE]-complex and anti-CD3. C) Activation of NFκB-luciferace reporter gene in J.RT3-T3-5 cells expressing TRBV7-9 by Caki-2 cells and different concentrations of ABR-217620 (squares) or SEA/E-120 (triangles). Activation of the NFκB-luciferace reporter without ABR-217620/SEA/E-120 was equal to the defined 0-value in the graph. D) Activation of NFκB-luciferace reporter gene in J.RT3-T3-5 cells expressing TRBV7-9 by 5T4- and anti-CD2-coated (squares) or control-coated (triangles) beads and different concentrations of ABR-217620. Activation of the NFκB-luciferace reporter without ABR-217620 was equal to the defined 0-value in the graph.

We recently reported that ABR-217620 induced killing of human 5T4^+^ tumor cells *in vitro* by human SEA and SEA/E-120 activated T lymphocytes [29]. This Sag antibody dependent cell-mediated cytotoxicity (SADCC) is strictly dose-dependent (99.7% of chromatographed sample represented monomeric ABR-217620 as depicted in Figure S4) and antigen selective depending on the antibody specificity of ABR-217620 [29]. Here we used SEA stimulated T cells, sorted the multimeric ABR-217620 binding subset by flow cytometry and evaluated the ABR-217620 SADCC against human 5T4^+^ MHC class II^-^ Caki-2 tumor cells. The sorted cells were cultured over night before being used as cytotoxic effector cells. The highly enriched (99%) multimeric ABR-217620 binding T cells (Figure 3A) showed an increased killing capacity in the SADCC assay as compared to the unsorted T cell subset (Figure 3B). No killing was observed when effector and target cells were incubated without ABR-217620. Thus, the TRBV7-9 expressing T cells are the predominant CTL type acting in the ABR-217620 mediated SADCC. 

### ABR-217620 activates TRBV7-9-transfected J.RT3-T3.5 cells in the presence of bead bound 5T4.

ABR-217620 has a low MHC class II binding capacity that potentially plays a role in the activation process of the TRBV7-9 expressing CTLs *in vivo* (Hedlund et al., to be published). The target Caki-2 cells are MHC class II^-^ but activated human T cells express MHC class II products [21]. To create a homogenous MHC class II^-^ system to further strengthen the MHC class II product independency of ABR-217620 in activation of T cells expressing TRBV7-9, the TCR β-chain- and MHC class II-deficient ([47] and Figures S5 and S6) T cell line J.RT3-T3.5 was employed. These cells lack surface expression of the TCR/CD3 complex. Transfection of J.RT3-T3.5 cells with an expression vector containing full-length TRBV7-9 chain reconstituted the CD3 expression on the cells surface (Figure S5). Expression of TRBV7-9 was detected on the transfected cells by FACS when stained with the multimeric ABR-217620 (Figure S7). The TRBV7-9^+^ J.RT3-T3.5 cells also contained an NFκB responsive luciferase reporter gene to allow detection of TCR-mediated activation.

The TRBV7-9-expressing J.RT3-T3.5 reporter cells were tested for reactivity against the Caki-2 cells in the presence of ABR-217620. Caki-2 cells incubated with ABR-217620 effectively stimulated NFκB signalling in the TRBV7-9^+^ J.RT3-T3.5 reporter cells in a concentration-dependent manner (Figure 3C). Furthermore, non-conjugated SEA/E-120 was unable to activate the reporter cells, demonstrating the importance of bridging the Caki-2 cells and the J.RT3-T3.5 reporter cells through the 5T4 antigen and the TRBV7-9 to activate NFκB. This is further supported by the fact that no NFκB activity was detected in cell cultures containing ABR-217620 in the absence of Caki-2 cells (Figure S8).

Next, the TRBV7-9^+^ J.RT3-T3.5 reporter cells were tested for reactivity against naked beads or beads coated with the 5T4 antigen and anti-CD2 Ab in the presence of ABR-217620. The 5T4 coated beads incubated with ABR-217620 effectively stimulated NFκB signaling in the TRBV7-9 expressing J.RT3-T3.5 reporter cells in a concentration-dependent manner (Figure 3D). The naked beads gave no stimulus either with or without ABR-217620. The data clearly indicate that the TRBV7-9 expressing T reporter cells are activated in a MHC class II-independent manner by 5T4-cell or -bead bound ABR-217620.

### ABR-217620 binds to 5T4 with high affinity, to TRBV7-9 with low affinity and to MHC class II with very low affinity

The conceptual objective for developing ABR-217620 was to selectively coat tumor cells with CTL target structures functionally similar to natural CTL pMHC target molecules. Therefore ABR-217620 contains a high affinity tumor binding Fab and an engineered Sag to engage T cells when bound to a tumor cell surface membrane. The Sags SEA and SEE bind to MHC class II products and the MHC class II/Sag complex efficiently activates T lymphocytes. SEA/E-120 has been modified to minimize MHC class II binding and therefore ABR-217620 has a comparably low capacity to activate T cells in a MHC class II dependent manner [29].

**Figure 4:**
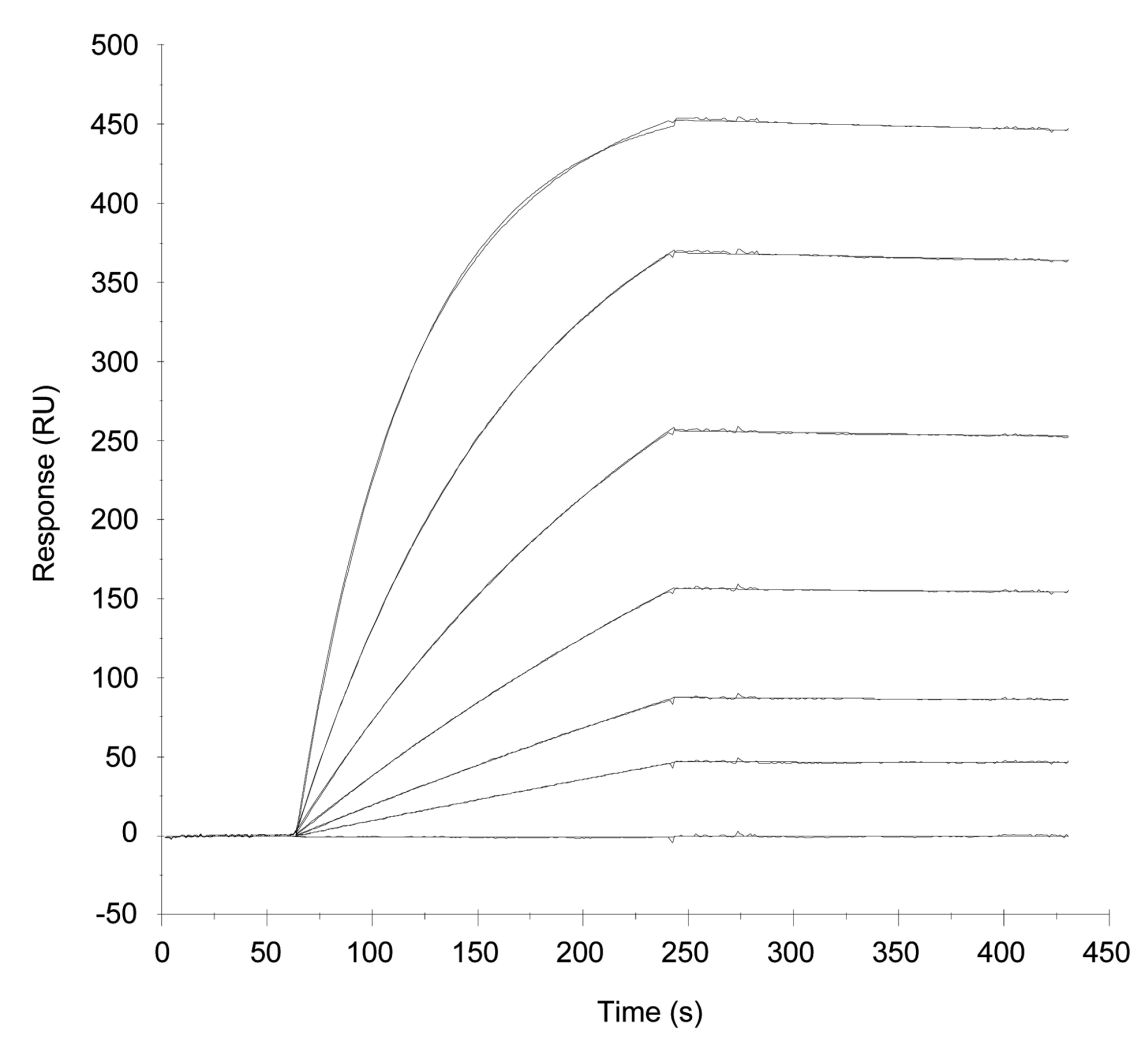
Surface plasmon resonance analysis of ABR-217620 binding to 5T4. ABR-217620 was injected for 3 min at a flow rate of 20 µL/min over 5T4Fc (density 770 RU). Regeneration of the surface was made by injecting 10 µL 10 mM glycine-HCl, pH 1.5, during 30 s. Sensorgrams from bottom to top represent sample buffer and ABR-217620 in the concentration range 1.56-50 nM. An affinity of 2.1 × 10^-10^ M was calculated after kinetic analysis of binding data by fit of sensorgrams to a 1:1 model with on- and off-rates of 3.6 × 10^5^ (1/Ms) and 7.5 × 10^-5^ (1/s).

To study binding to tumor antigen, ABR-217620 was injected over a surface with 5T4 antigen covalently coupled on a biosensor chip. Dose-dependent and specific binding was demonstrated for ABR-217620. Sensorgrams in Figure 4 were obtained after injection of ABR-217620 in the concentration range 1.56-50 nM. Kinetic analysis was performed by fit of sensorgrams to a 1:1 model. A K_D_ of ~ 0.2 nM was calculated for this interaction while no binding was obtained with SEA/E-120 fused with C215Fab (Figures S9 and S10). Hence, the high picomolar affinity for the 5T4 antigen efficiently directs ABR-217620 to this tumor associated antigen. 

**Figure 5:**
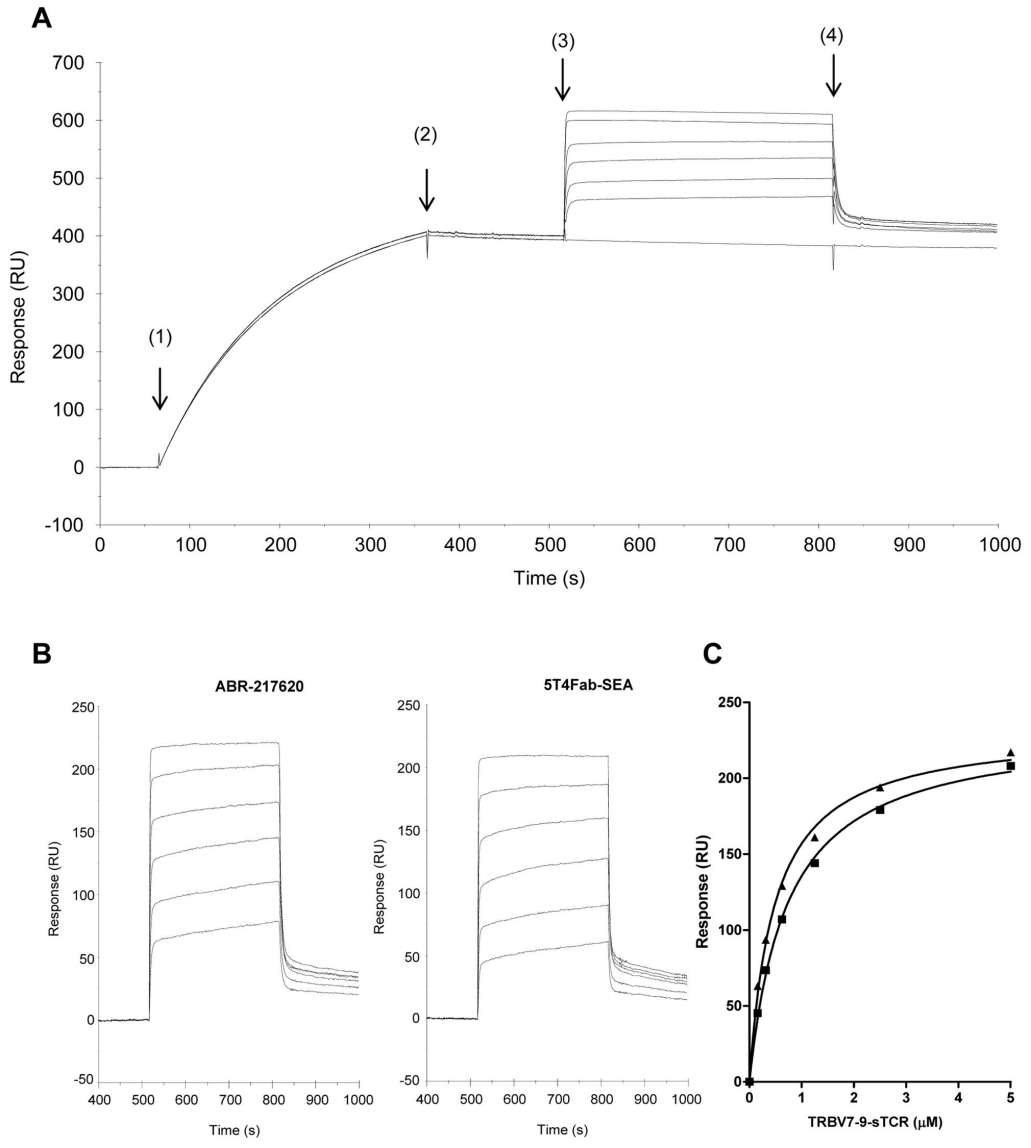
Surface plasmon resonance analysis of TRBV7-9 interaction with ABR-217620 or 5T4Fab-SEA. 25 nM ABR-217620 or 5T4Fab-SEA was captured (5 min at 20 µL/min) on immobilized 5T4Fc (density ~ 990 RU) prior to injection (5 min at 20 µL/min) of 0.156-5 µM TRBV7-9. The surface was regenerated with a short 10 µL pulse of 10 mM glycine-HCl, pH 1.5. A) Sensorgrams obtained after injection of 25 nM ABR-217620 (1) followed by 0-5 µM TRBV7-9 (3) with buffer pumped over the surface at (2) and (4). B) Injection of 0.156-5 µM TRBV7-9 over ABR-217620 and 5T4Fab-SEA after subtraction of sensorgram with sample buffer without TRBV7-9. C) Responses at early association phase (t ~ 530 s) plotted versus TRBV7-9 concentration for binding to captured 5T4Fab-SEA (squares) and ABR-217620 (triangles). Curves were fit to a one-site hyperbola model in GraphPad Prism for calculation of maximal response and apparent affinity.

In a next step we wanted to assess the interaction between the TCR and the Sag moiety in ABR-217620. For this purpose, 25 nM ABR-217620 or 5T4Fab-SEA was captured on 5T4Fc immobilized at a relatively low density prior to injection of TRBV7-9 at a concentration ranging from 0.156 to 5 µM. The resulting sensorgrams for TRBV7-9 binding to captured ABR-217620 are shown in Figure 5A demonstrating fast “box-shaped” kinetics. In Figure 5B binding of TRBV7-9 to both 5T4Fab-SEA and ABR-217620 is shown after subtraction of sensorgram obtained with TRBV7-9 replaced by sample buffer as a second injection. Responses were calculated immediately after injection of TCR (t ~ 530 s) and plotted against soluble TCR concentration (Figure 5C). Curves were fit to a one-site hyperbola model in GraphPad Prism for calculation of B_max_ and apparent affinity. Response maximum and apparent affinity were found to be similar for the binding of TRBV7-9 to SEA and SEA/E-120 (Table 1). The control TCR containing TRBV6-5 was not able to interact with captured SEA/E-120 (Figure S11).

**Table 1:** Summary kinetic analysis of sTRBV7-9 to SEA, SEE and SEA/E-120 Fab fusion proteins.

	Captured fusion protein				
SAg moiety	SEA		SEE	SEA/E-120	
Fab moiety	5T4	C215	C215	5T4^†^	C215
Capture level (RU)	394	109	134	401	133
B_max_ (RU)	234 + 5	53.2 + 1.5	53.3 + 2.4	232 + 5	75.7 + 1.3
K_D_ (µM)	0. 73 + 0.04	1.2 + 0.1	6.1 + 0.4	0.48 + 0.04	0.58 + 0.03

**Figure 6:**
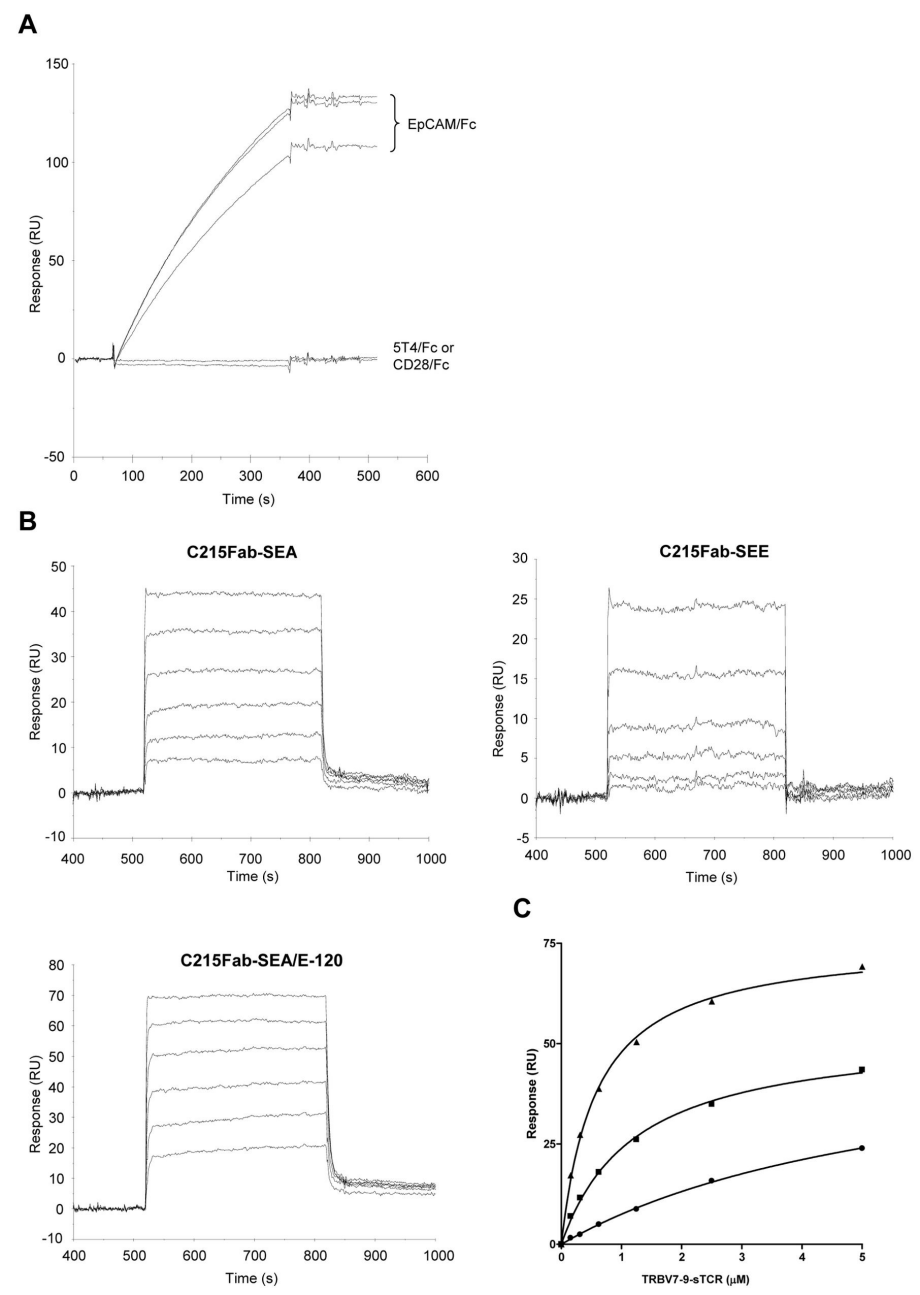
Surface plasmon resonance analysis of TRBV7-9 binding to fusion proteins containing SEA, SEE and SEA/E-120. A) Sensorgrams obtained after injection (5 min at 20 µL/min) of 25 nM C215Fab-SEA, -SEE and –SEA/E-120 over amine coupled EpCAM/Fc (density ~ 870 RU). Capture levels of 109, 134 and 133 were calculated for C215Fab-SEA, –SEE and –SEA/E-120 respectively. No binding was observed when these samples were injected over CD28/Fc (density ~ 680 RU) or 5T4Fc (density ~ 990 RU). B) Sensorgrams showing binding of 0.156 to 5 µM TRBV7-9 to EpCAM captured C215Fab-SEA, C215-SEE or C215–SEA/E-120 after subtraction of sensorgram with sample buffer. C) Response levels at t ~ 530 s plotted against TRBV7-9 concentration for binding to SEA (squares), SEE (circles) and SEA/E-120 (triangles) fusion proteins. Curves were fit to a one-site hyperbola model in GraphPad Prism for calculation of maximal response and apparent affinity.

Curves were fit to a one-site hyperbola model in GraphPad Prism (see Figures 5C and 6C). Values are given as mean + SE.

^†^ ABR-217620

In an additional series of experiments, SEA, SEE and SEA/E-120 fused with a Fab recognizing human EpCAM or C215 [14,34] were bound to EpCAMFc immobilized at a low density. As is shown in Figure 6A, the C215Fab fusion proteins were bound with high affinity to the EpCAM surface with very low dissociation prior to injection of sTCR. Surfaces with 5T4Fc and CD28Fc were used as specificity controls and demonstrated no binding of Sags fused with C215Fab. Serially diluted TRBV7-9 was then injected over EpCAM captured C215Fab-SEA, -SEE and –SEA/E-120 (Figure 6B) demonstrating kinetics similar to that observed for 5T4Fab-Sags. In Figure 6C, responses at steady-state levels were plotted against TRBV7-9 concentration and with curves fit as described in Figure 5C. TRBV7-9 demonstrated a more than ten-fold lower affinity for SEE than SEA/E-120 and a nearly six-fold lower affinity compared to SEA. Results from both TCR binding experiments are summarized in Table 1. 

**Figure 7:**
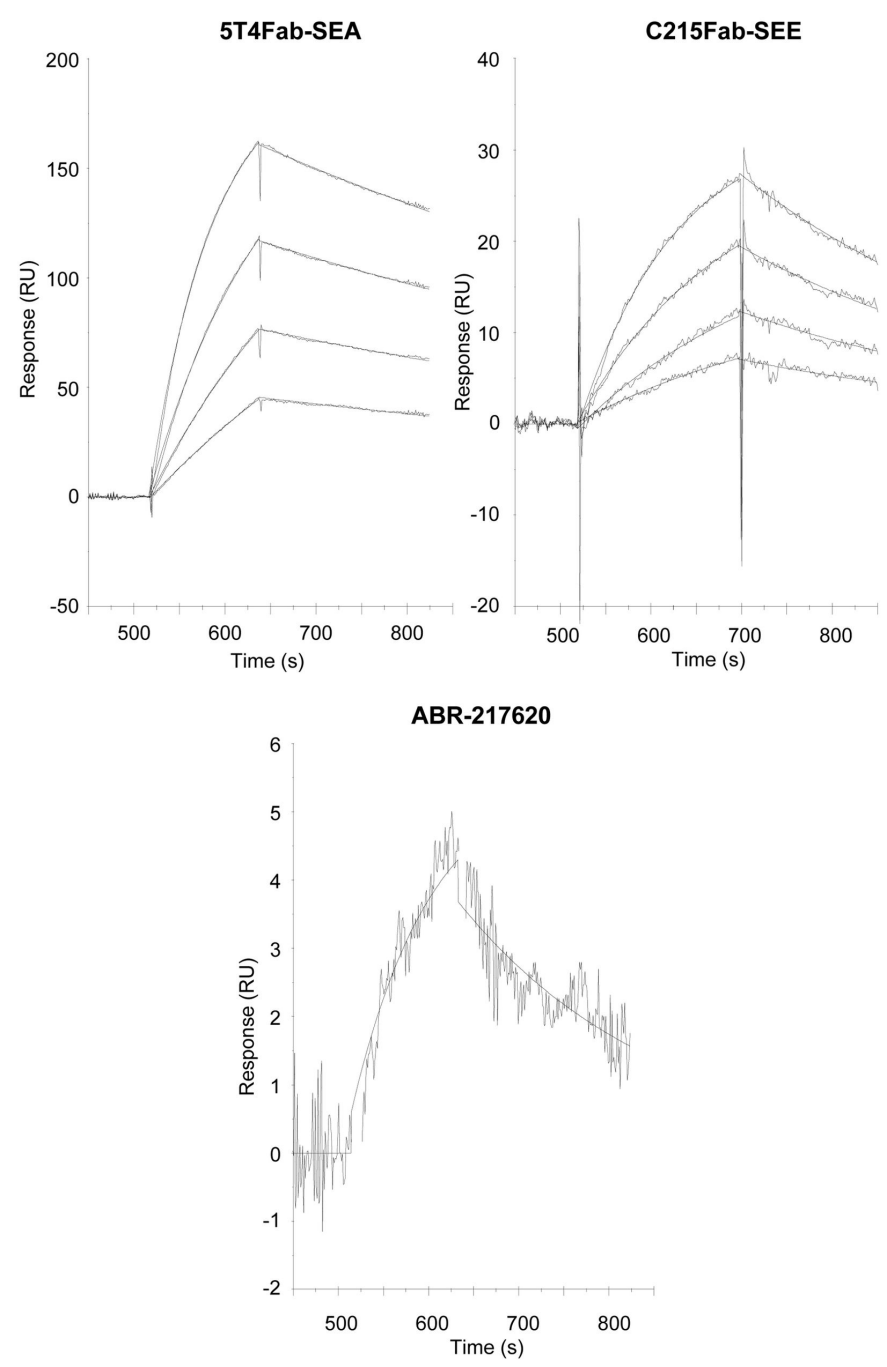
Surface plasmon resonance analysis of HLA-DR1 HA peptide complex binding to antigen captured SEA, SEE and SEA/E-120. 50 nM fusion proteins containing SEA, SEE and SEA/E-120 were captured (5 min at 20 µL/min) on either immobilized 5T4Fc (ABR-217620 and 5T4Fab-SEA) or EpCAMFc (C215Fab-SEE) prior to injection (2 min at 20 µL/min) of 0.625-5 µM HLA-DR1/HA (mol wt ~ 44.7 K). Sensorgrams show binding of HLA-DR1/HA to captured 5T4Fab-SEA, C215Fab-SEE and ABR-217620 after subtraction of SPR signal obtained when sample buffer (HBS-P containing 10 µM Zn^2+^) was injected over captured Fab fusion proteins. For ABR-217620 only binding of the HLA-DR1/HA complex at 5 µM is shown as the signal at the lower concentrations was too low for accurate kinetic evaluation.

An extremely weak affinity was anticipated for the ABR-217620 interaction with MHC class II products as SEA/E-120 was subject to multiple mutations resulting in low cell binding activity *in vitro*. In this study, we have used recombinant human HLA-DR1 in complex with an HA antigen peptide to study binding using the SPR technology. This technology allows probing of affinities as weak as in the high micromolar range in a real-time heterogeneous format. Fusion protein containing SEA, SEE and SEA/E-120 were captured at 50 nM on either immobilized 5T4Fc (ABR-217620 and 5T4Fab-SEA) or EpCAMFc (C215Fab-SEE) prior to injection of 0.625-5 µM HLA-DR1/HA. Figure 7 shows binding of HLA-DR1/HA to captured Sags after subtraction of sensorgram obtained when injecting sample buffer (HBS-P containing 10 µM Zn^2+^) in the absence of HLA-DR1/HA. Sensorgrams were fit to a 1:1 model in BIAevaluation for kinetic analysis. Dose-dependent binding was demonstrated to both SEA and SEE with affinities in the high nanomolar to low micromolar range. In contrast, HLA-DR1/HA binding to antigen captured SEA/E-120 was only clearly distinguishable from the baseline first at the highest concentration. Therefore, kinetic analysis was only made with the sensorgram obtained at 5 µM indicating a 130 and 40 times lower affinity of HLA-DR1/HA for SEA/E-120 than for SEA and SEE. This lower affinity of SEA/E-120 for MHC class II is primarily driven by a loss of on-rate which was 29 and 24 times lower than for SEA and SEE. Evaluation of HLA-DR1/HA binding data is summarized in Table 2. 

**Table 2:** Kinetic analysis of HLA-DR1/HA complex binding to fusion proteins containing SEA, SEE and SEA/E-120.

	Captured fusion protein		
	5T4Fab-SEA	C215Fab-SEE	ABR-217620^†^
Capture level ^‡^ (RU)	505	93.4	507
k_on_ (1/Ms)	2.8×10^3^	2.3×10^3^	9.5×10^1^
k_off_ (1/s)	1.1×10^-3^	2.9×10^-3^	4.8×10^-3^
K_D_ (µM)	0.4	1.3	51
R_max_ (RU)*	215	37.5	7.9
Chi^2^	0.08	0.19	0.11

Sensorgrams were fit to a 1:1 model in BIAevaluation for kinetic analysis.

^†^ only sensorgram with 5 µM HLA-DR1/HA used for kinetic analysis

^‡^ level prior to injection of HLA-DR1/HA (t ~ 500 s)

* calculated from R_eq_ values plotted against HLA-DR1/HA concentrations

In summary, surface plasmon resonance experiments have revealed binding of ABR-217620 to 5T4, TRBV7-9 and HLA-DR1 with very different affinities, which equals to 0.2 nM, 0.5 µM and 51 µM, respectively, dictating its pharmacology and selective immune activities.

## Discussion

The conceptual objective for developing ABR-217620 was to selectively coat tumor cells with CTL target structures functionally similar to natural CTL pMHC target molecules. Here we present data showing that the molecular basis for at least part of the anti-tumor activity by ABR-217620 resides in the distinct interaction between the T lymphocyte membrane protein TRBV7-9 and the Sag SEA/E-120 in the fusion protein bound to the 5T4 antigen on tumor cells. The T lymphocyte engagement by ABR-217620 is obtained by its high affinity binding to tumor cells (K_D_ approximately 1 nM) and the mimicry of natural productive immune TCR-pMHC contact using affinities around 1 µM.

Efficient targeting of the therapeutic fusion protein ABR-217620 to 5T4 expressing tumor cells is probably dependent on a highly selective high affinity binding to the 5T4 oncofetal antigen. We have previously estimated the affinity of ABR-217620 binding to 5T4 expressing tumor cells *in vitro* to be 1.6 nM [29]. Here we show using the SPR technology that the affinity to the 5T4 antigen is approximately 0.2 nM. This confirms the affinity between ABR-217620 and the 5T4 antigen to be around 1 nM.

Wild type Sags (wtSags) bind to TCRs with low to very low affinities (K_D_ typically 1-100 µM) and are presented by antigen presenting cells (APCs) through their binding to MHC class II molecules [44,48,49]. The high T lymphocyte activation capacity by Sags is a result of an evolutionary process balancing the binding affinities to the T cells and the APCs as mimicry of the TCR-pMHC interaction. The binding of wtSags to MHC class II proteins is the prerequisite for T cell activation, toxicity and the capacity to direct CTLs in Sag-dependent cell-mediated cytotoxicity (SDCC) [50]. As shown here and previously [29], the targeting of CTLs by ABR-217620 to tumor cells in the SADCC reflects the high affinity binding to the 5T4 tumor associated antigen. The very low affinity of ABR-217620 for MHC class II proteins results in drastically reduced toxicity as compared to classic wtSags [26,27]. Low efficiency MHC class II protein binding is probably advantageous to achieve reproducible productive TH1 responses with large numbers of CTLs and increased tumor exposure. We cannot conclude from the present data if the MHC class II protein binding can be totally abolished with retained anti-tumor effects, despite having shown that the CTL effector phase is independent of MHC class II expression. Preliminary data from TRBV7-9 / HLA-DR4 single and double transgenic mice indicate the necessity of HLA-DR4 expression in addition to TRBV7-9 to achieve maximal antitumor activity from ABR-217620 treatment (Hedlund et al., to be published). In line with these observations, we show here that ABR-217620 binds to 5T4, TRBV7-9 and human MHC class II (HLA-DR) and most importantly with very different affinities dictating its pharmacology and selective immune activities. Binding to CD28 as shown with free Sags by Arad et al. [51] could be of potential impact on the integrated immune activities induced by ABR-217620. However, no binding to CD28 was detected with Sag containing fusion proteins (Figure 6A). 

Interestingly, SEA/E-120 has an extremely narrow TCRVβ profile, with great preference for TRBV7-9. An explanation for this most likely resides on both the TCR side as well as on the MHC class II binding side of the Sag molecule. When removing the high-affinity MHC class II binding site of the Sag, a narrowing of the Vβ repertoire was shown for the SEA mutant D227A, which is in line with the observed narrowing shown here for SEA/E-120. Furthermore, much of the TCRVβ restriction is probably inherited from SEE (Figure 2).

Aleksic et al. concluded that TCR-pMHC confinement time governs productive T cell activation [52]. In cases with fast on-rates and fast off-rates resulting in short dwell time as measured in 3D systems, rebinding in the cell plasma membrane 2D systems adds up to sufficient confinement time and productive activation [52,53]. Despite that the 5T4 tumor antigen may be expressed in high density on tumor cells *in vivo*, the density of tumor cell bound ABR-217620 is expected to be variable in tumor tissue and probably very low in certain areas. An optimal productive T cell activation signal can be achieved with TCR-pMHC interactions with affinities around 1 µM and with few pMHC per target cell [9,12]. Here we show that ABR-217620 selectively binds to TRBV7-9 with an affinity of approximately 1 µM and with a binding pattern allowing dissociation and rebinding. ABR-217620 induces killing of 5T4^+^ tumor cells by CTLs expressing TRBV7-9. TTSs containing wtSags SEA and SEE have been shown to activate T cells but only fusion proteins containing SEA could induce SADCC [54]. SEE showed a much lower affinity for TRBV7-9 as compared to SEA but SEE can efficiently activate TRBV7-9^+^ T cells when bound to MHC class II proteins. It may be speculated that only Sags with TCR-affinities above a certain level trigger CTLs to kill tumors when using TTSs. On the other hand, it would be counter-productive to increase the affinity above a certain level and thereby also inducing T cell binding in free solution.

The single-chain bispecific T cell engaging antibody concept (BiTE) [55] is similar to the TTS concept in that it also involves redirection of CTLs to tumors by an anti-tumor antibody moiety with high affinity. An interesting variation on this theme is realized in monoclonal TCR-redirected tumor cell killing [56]. In this concept, high affinity anti-tumor TCRs are fused to an anti-CD3 moiety. Similar to TTS, the CD3-targeted bispecific fusion proteins show large differences in affinity between binding to tumor antigen and to activator protein on T cells with preference for the tumor. 

The very low affinity but probably biologically important MHC class II binding distinguishes ABR-217620 from other bispecific fusion proteins. A major objective in the development of ABR-217620 has been to acquire high tumor targeting capability and a short general exposure to gain maximal effects in the tumor without general toxicity seen with e.g. wild-type SEA [27]. The mean terminal half-life was determined to be around 1 h [27]. This short half-life is typical also for the BiTE single-chain proteins [55]. No time-dependency of ABR-217620 was seen in the pharmacokinetics in treatment cycle 1 with similar plasma concentrations on day 1 and day 5. During treatment cycle 1 no significant dependency in the pharmacokinetic parameters was seen due to anti-SEA/E-120 antibody levels. The anti-SEA/E-120 levels were stable or moderately increased in approximately 50% of the patients after 1 cycle. In general systemic exposure of ABR-217620 was lower in treatment cycle 2 and 3, presumably from induced antibodies [26]. 

Another important difference compared to ABR-217620 might be that CD3-targeted bispecific fusion proteins typically bind to CD3 with affinities higher than those of ABR-217620 to TRBV7-9. While monomeric binding of bispecific T cell engaging antibodies to CD3 on T cells was shown [57], ABR-217620 has insufficient affinity to allow for significant monomeric T cell binding. ABR-217620 shows T lymphocyte binding requirements similar to soluble pMHC staining only as multimers [10]. Our findings implicate that tumor cells with high affinity captured TTS fusion protein on their surface interact with TCRs with kinetics that efficiently trigger activated CTLs (i.e. fast on- and off-rates and a K_D_ of around 1 µM). 

ABR-217620 has shown proof of principle in preclinical immune pharmacology and tumor models as well as in clinical studies [26,29]. It induces selective immune activation and targets T lymphocytes to 5T4 expressing tumors. Promising results in RCC and NSCLC patients with prolonged overall survival were recorded in clinical phase 1 and phase 2a trials using ABR-217620 and its predecessor ABR-214936, respectively [24,26,27]. ABR-217620 is presently in phase 2/3 clinical trials in combination with IFN-α for treatment of RCC. In this study we show that ABR-217620 has the capacity to bind, selectively activate and retarget a large proportion of T cells, the TRBV7-9 expressing T cells, in a fashion very similar to how CTLs are engaged to kill when recognizing their target pMHC on a cell surface. At the same time ABR-217620 avoids binding, blocking and possibly triggering as monomeric target structure in body fluids. 

## Supporting Information

Figure S1 **Schematic figure depicting different fusion proteins.** The Tumor Targeted Superantigens are naptumomab estafenatox, 5T4-SEA, C215-SEA/E-120, C215-SEA and C215-SEE. 5T4Fab, Fab recognizing the oncofetal antigen 5T4; C215Fab, Fab recognizing EpCAM; SEA, staphylococcal enterotoxin A; SEA/E-120, engineered hybrid of staphylococcal enterotoxin A and E; SEE, staphylococcal enterotoxin E.
(TIF)

Figure S2 **Schematic figure depicting the basic targeting and anti-tumor principles of Tumor Targeted Superantigens (TTS).** IFN, Interferon; TAA, Tumor associated antigen; TCR, T cell receptor; TNF, Tumor Necrosis Factor.
(TIF)

Figure S3 **Flow cytometry analysis of binding of the [ABR-217620-Biotin/SA-PE]-complexes, consisting of ABR-217620-biotin and SA-PE at different molar ratios (ABR-217620-biotin:SA-PE), to a SEA-reactive T cell line.**
(TIF)

Figure S4 **Size-exclusion chromatography of ABR-217620 revealed no tendency to form aggregates.** 30 µg ABR-217620 was injected on a Superdex 200 HPLC column (300 x 10 mm; GE Healthcare) and eluted with 150 mM citric acid, pH 5.0, at a flow rate of 0.35 mL/min at 25°C. The monomer and dimer peaks were eluted at 41 and 38 min respectively and constituted 99.8 and 0.2%. No peak was visible at the position of aggregates (23.5 min). Protein was monitored using a fluorescence detector.
(TIF)

Figure S5 **Flow cytometry analysis of CD3 expression on J.RT3-T3-5 wild type and J.RT3-T3-5 cells expressing TRBV7-9 (control is dotted).**
(TIF)

Figure S6 **Flow cytometry analysis of MHC class II expression on J.RT3-T3-5 cells expressing TRBV7-9 (control is dotted).**
(TIF)

Figure S7 **Flow cytometry analysis of [ABR-217620-Biotin/SA-PE]-complex binding to J.RT3-T3-5 wild type and J.RT3-T3-5 cells expressing TRBV7-9 (control is dotted).**
(TIF)

Figure S8 **Activation of NFκB-luciferace reporter gene in J.RT3-T3-5 cells expressing TRBV7-9 by different concentrations of ABR-217620 in the absence (open) and presence (filled) of Caki-2 cells.**
(TIF)

Figure S9 **ABR-217620 demonstrates high-affinity and specific binding to the 5T4 antigen.** Sensorgrams obtained after injection (5 min at 20 µL/min) of 25 nM ABR-217620 or 5T4FabSEA over recombinant 5T4, EpCAM or CD28, fused with human IgG1Fc, and immobilized at similar densities (680 to 990 RU). Sample buffer (10 mM HEPES, 0.15 M NaCl, pH 7.4, containing 0.005% v/v Surfactant P20; HBS-P) was injected as a background control. Regeneration was carried out with 15 µL pulse of 10 mM glycine-HCl, pH 1.5.
(TIF)

Figure S10 **ABR-217620 demonstrates high-affinity and specific binding to the 5T4 antigen.** Sensorgrams obtained after injection of 6.25-50 nM SEA/E-120 fused with 5T4Fab (ABR-217620) or C215Fab. Samples were injected for 3 min at 20 µL/min over amine coupled rh5T4Fc (density ~ 2.5 kRU). Sample buffer and regeneration conditions were as in Figure S9.
(TIF)

Figure S11 **ABR-217620 demonstrates selective interaction with TRBV7-9.** Binding of TRBV7-9 and TRBV6-5 to ABR-217620. Samples were injected (2 min at 20 µL/min) over ABR-217620 (density ~724 RU) in the concentration range 0.0625-1 µM. The surface was regenerated by dissociation in running buffer. Only TRBV7-9 showed detectable binding to ABR-217620.
(TIF)
