# Supplementary figures and images for: The Tumor Targeted Superantigen ABR-217620 Selectively Engages TRBV7-9 and Exploits TCR-pMHC Affinity Mimicry in Mediating T Cell Cytotoxicity
Source: PLoS One. 2013 Oct 23;8(10):e79082. doi: 10.1371/journal.pone.0079082 (PMC3806850; doi:10.1371/journal.pone.0079082)

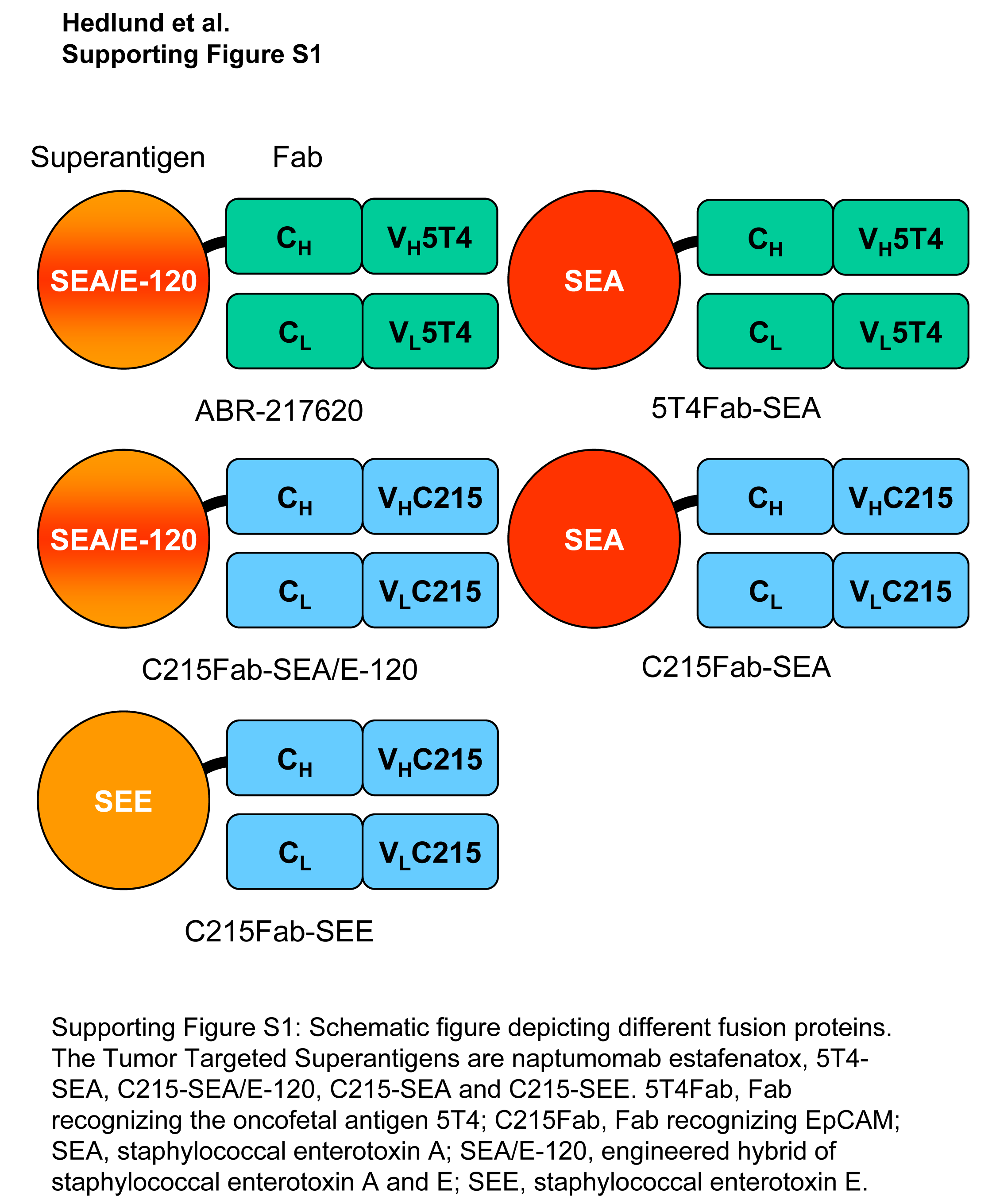

Supplement: Figure S1 — Schematic figure depicting different fusion proteins. The Tumor Targeted Superantigens are naptumomab estafenatox, 5T4-SEA, C215-SEA/E-120, C215-SEA and C215-SEE. 5T4Fab, Fab recognizing the oncofetal antigen 5T4; C215Fab, Fab recognizing EpCAM; SEA, staphylococcal enterotoxin A; SEA/E-120, engineered hybrid of staphylococcal enterotoxin A and E; SEE, staphylococcal enterotoxin E. (TIF) [file pone.0079082.s001.tif]

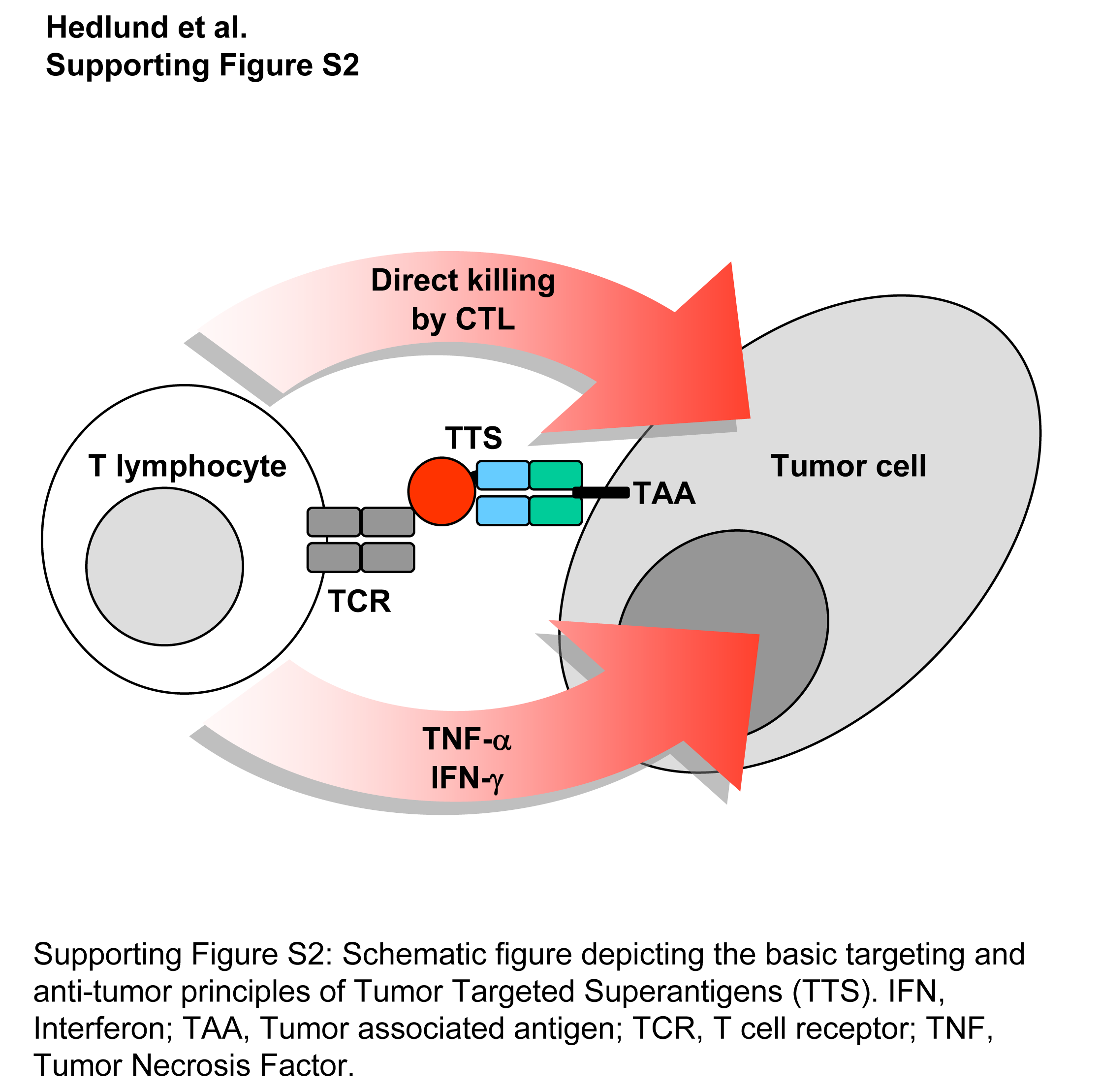

Supplement: Figure S2 — Schematic figure depicting the basic targeting and anti-tumor principles of Tumor Targeted Superantigens (TTS). IFN, Interferon; TAA, Tumor associated antigen; TCR, T cell receptor; TNF, Tumor Necrosis Factor. (TIF) [file pone.0079082.s002.tif]

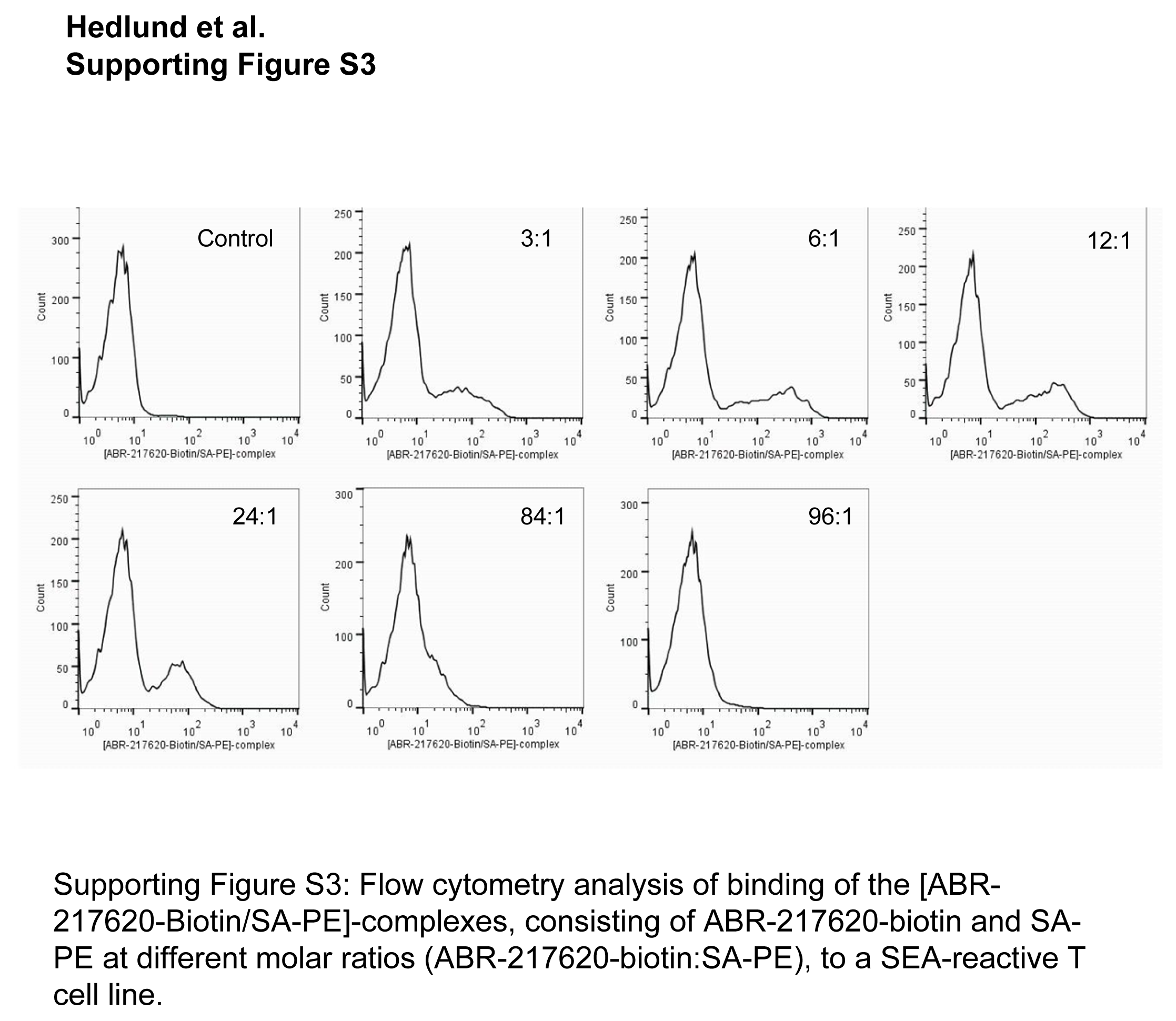

Supplement: Figure S3 — Flow cytometry analysis of binding of the [ABR-217620-Biotin/SA-PE]-complexes, consisting of ABR-217620-biotin and SA-PE at different molar ratios (ABR-217620-biotin:SA-PE), to a SEA-reactive T cell line. (TIF) [file pone.0079082.s003.tif]

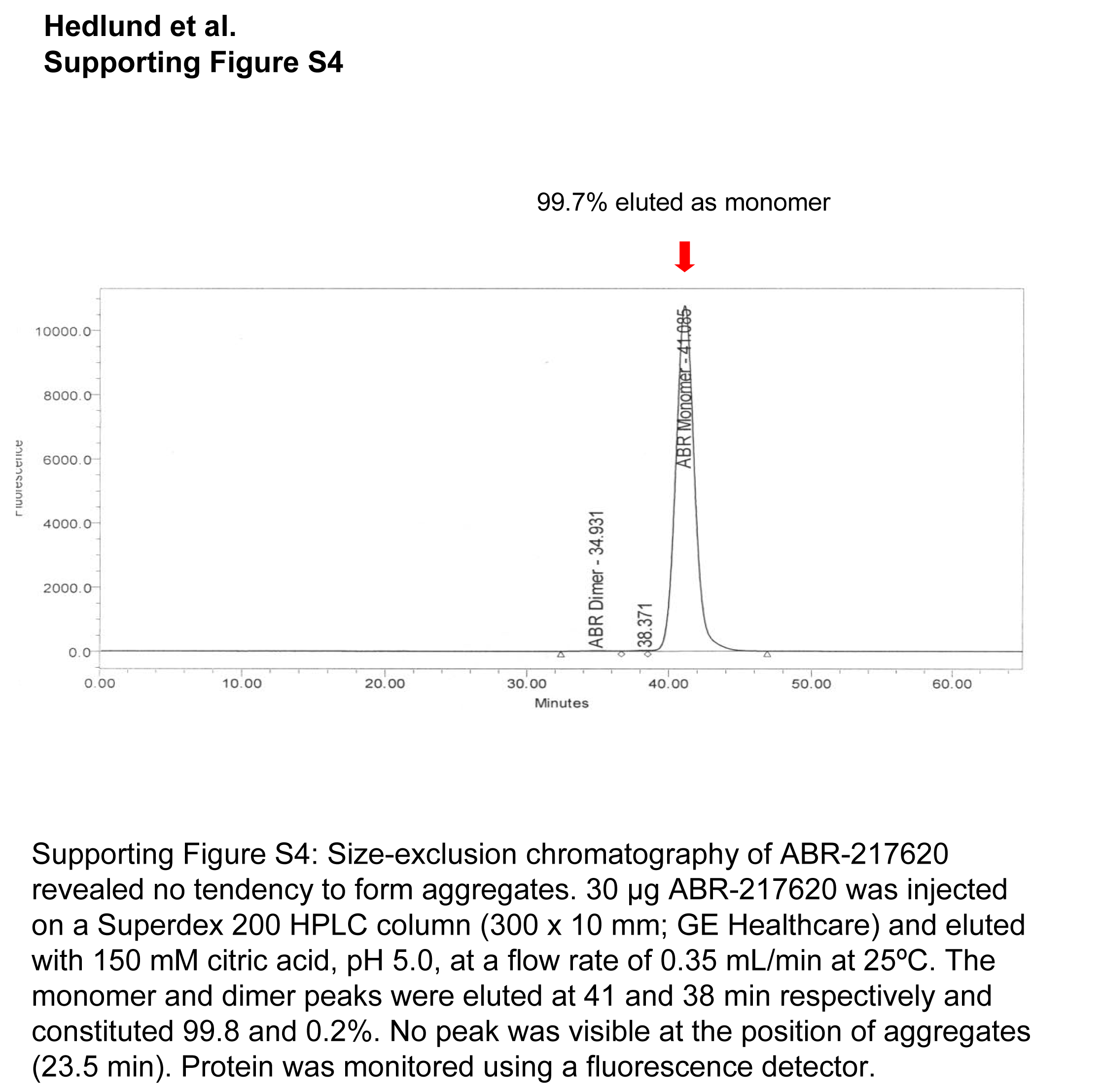

Supplement: Figure S4 — Size-exclusion chromatography of ABR-217620 revealed no tendency to form aggregates. 30 µg ABR-217620 was injected on a Superdex 200 HPLC column (300 x 10 mm; GE Healthcare) and eluted with 150 mM citric acid, pH 5.0, at a flow rate of 0.35 mL/min at 25°C. The monomer and dimer peaks were eluted at 41 and 38 min respectively and constituted 99.8 and 0.2%. No peak was visible at the position of aggregates (23.5 min). Protein was monitored using a fluorescence detector. (TIF) [file pone.0079082.s004.tif]

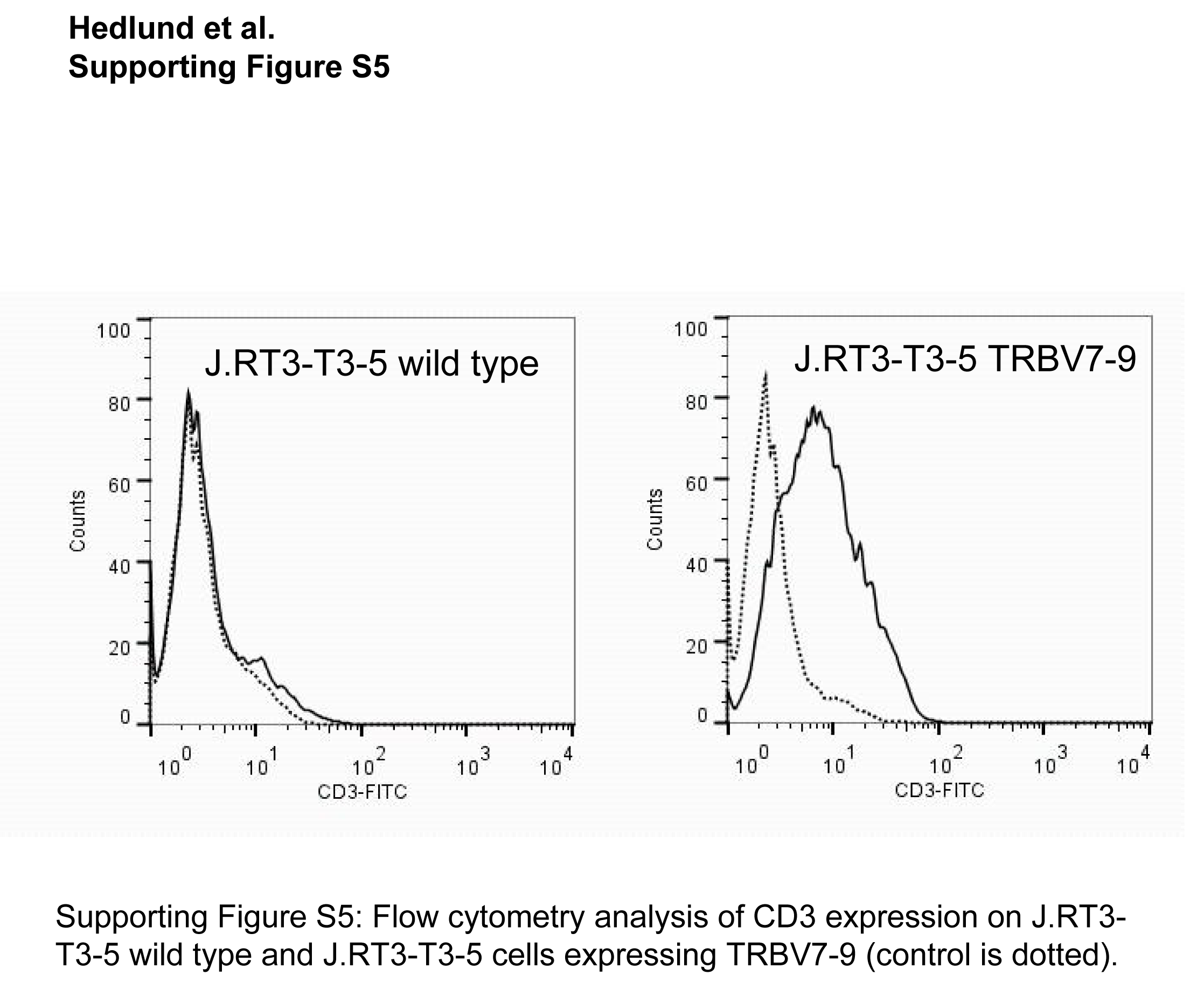

Supplement: Figure S5 — Flow cytometry analysis of CD3 expression on J.RT3-T3-5 wild type and J.RT3-T3-5 cells expressing TRBV7-9 (control is dotted). (TIF) [file pone.0079082.s005.tif]

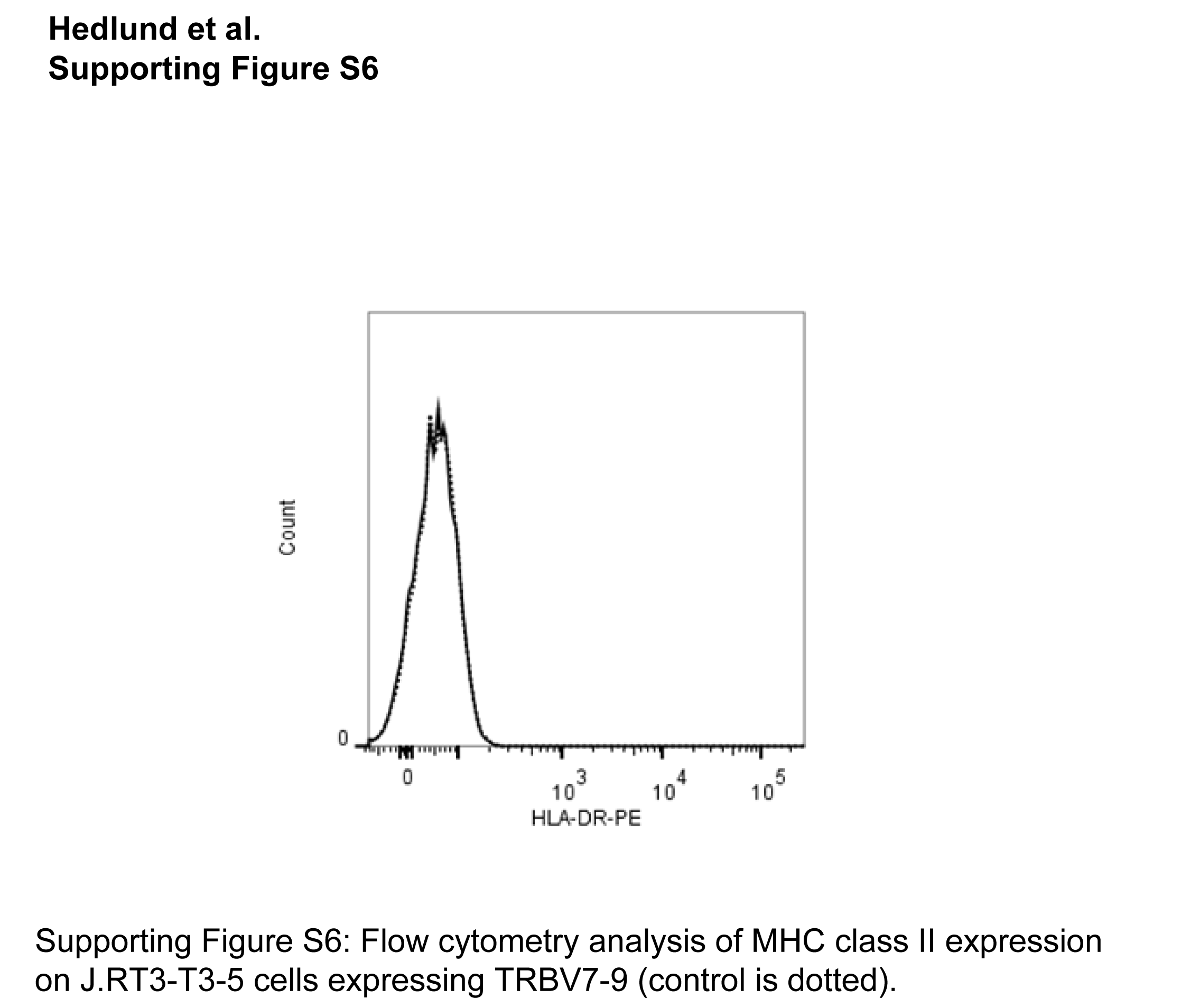

Supplement: Figure S6 — Flow cytometry analysis of MHC class II expression on J.RT3-T3-5 cells expressing TRBV7-9 (control is dotted). (TIF) [file pone.0079082.s006.tif]

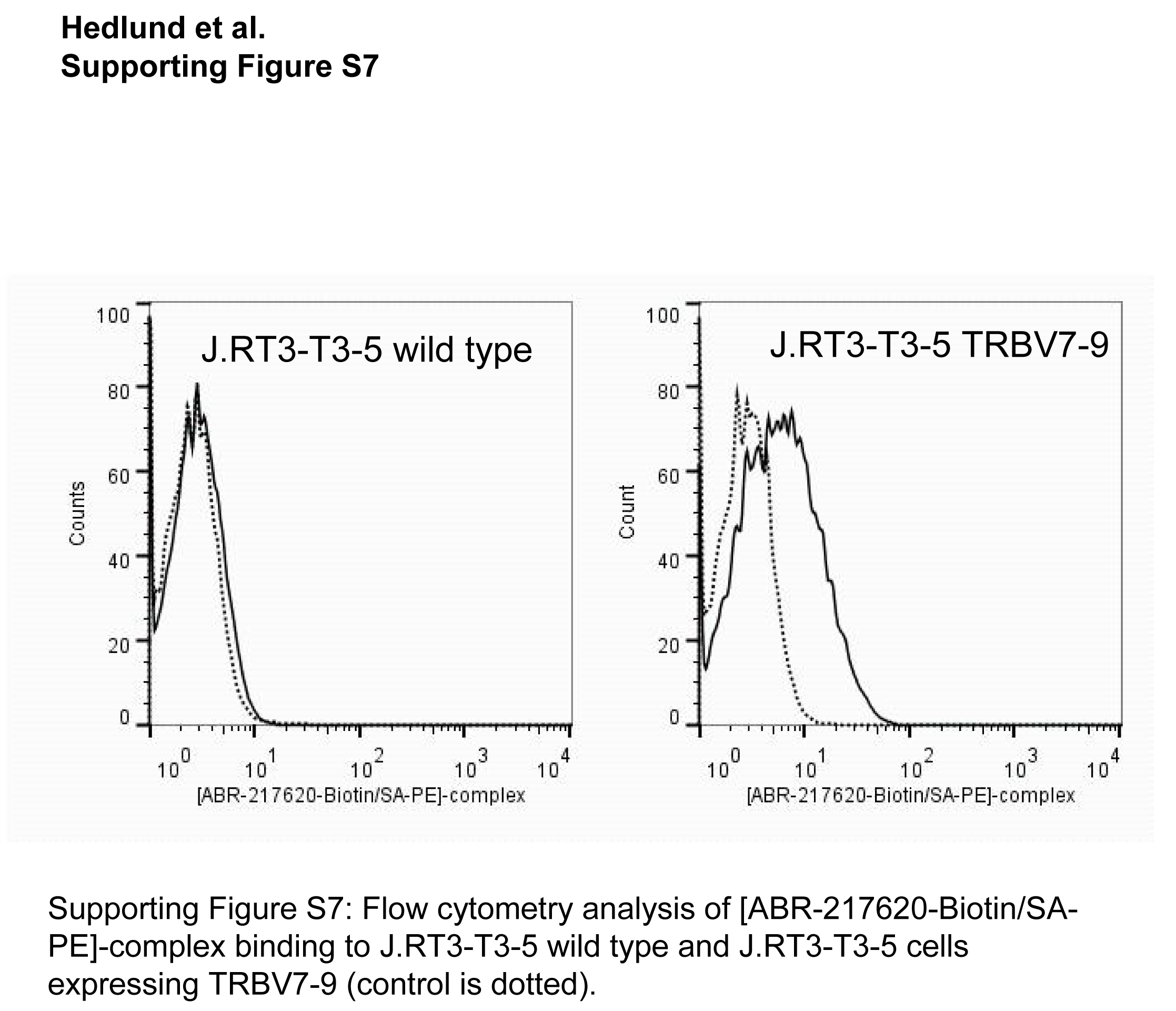

Supplement: Figure S7 — Flow cytometry analysis of [ABR-217620-Biotin/SA-PE]-complex binding to J.RT3-T3-5 wild type and J.RT3-T3-5 cells expressing TRBV7-9 (control is dotted). (TIF) [file pone.0079082.s007.tif]

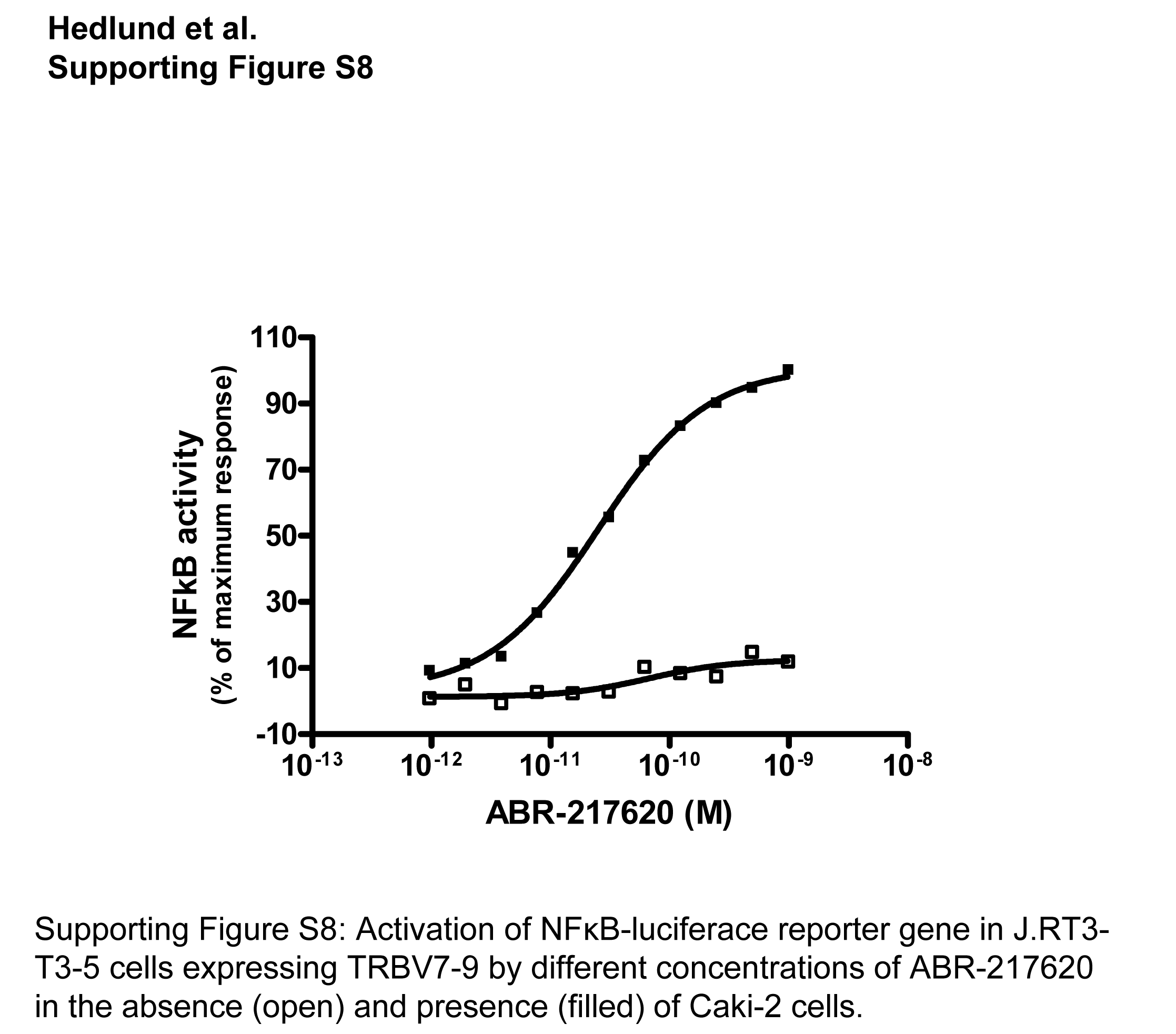

Supplement: Figure S8 — Activation of NFκB-luciferace reporter gene in J.RT3-T3-5 cells expressing TRBV7-9 by different concentrations of ABR-217620 in the absence (open) and presence (filled) of Caki-2 cells. (TIF) [file pone.0079082.s008.tif]

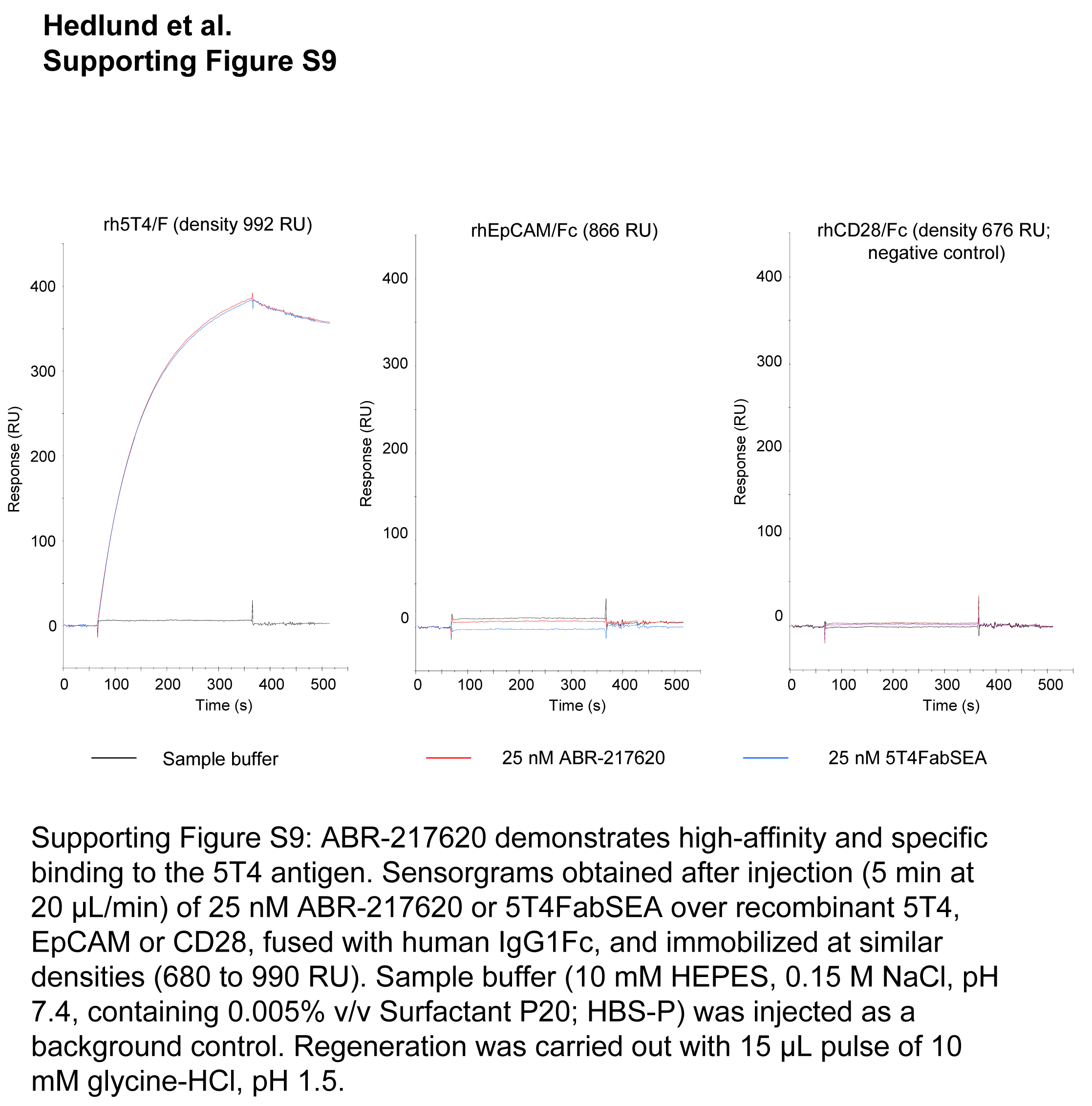

Supplement: Figure S9 — ABR-217620 demonstrates high-affinity and specific binding to the 5T4 antigen. Sensorgrams obtained after injection (5 min at 20 µL/min) of 25 nM ABR-217620 or 5T4FabSEA over recombinant 5T4, EpCAM or CD28, fused with human IgG1Fc, and immobilized at similar densities (680 to 990 RU). Sample buffer (10 mM HEPES, 0.15 M NaCl, pH 7.4, containing 0.005% v/v Surfactant P20; HBS-P) was injected as a background control. Regeneration was carried out with 15 µL pulse of 10 mM glycine-HCl, pH 1.5. (TIF) [file pone.0079082.s009.tif]

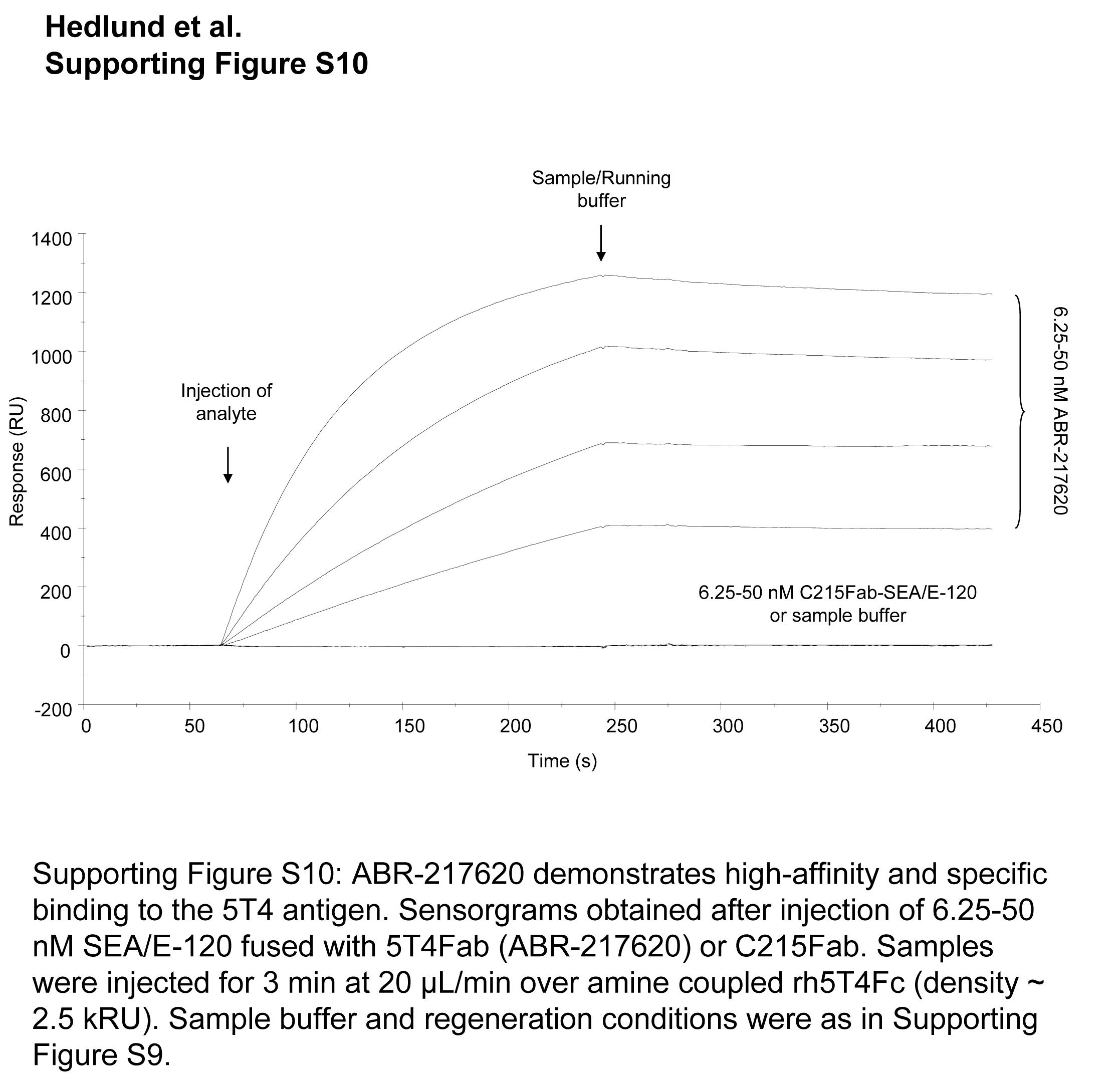

Supplement: Figure S10 — ABR-217620 demonstrates high-affinity and specific binding to the 5T4 antigen. Sensorgrams obtained after injection of 6.25-50 nM SEA/E-120 fused with 5T4Fab (ABR-217620) or C215Fab. Samples were injected for 3 min at 20 µL/min over amine coupled rh5T4Fc (density ~ 2.5 kRU). Sample buffer and regeneration conditions were as in Figure S9. (TIF) [file pone.0079082.s010.tif]

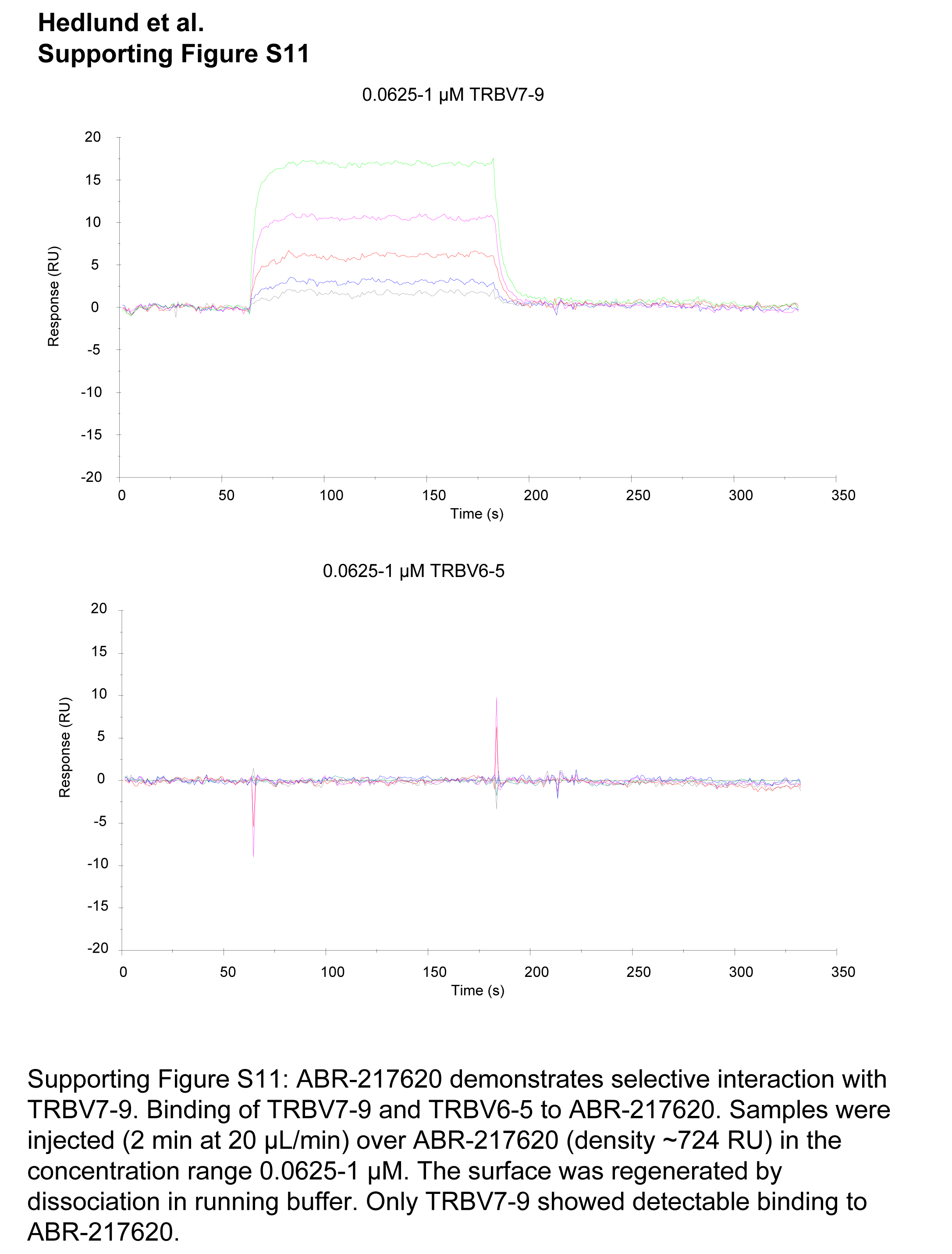

Supplement: Figure S11 — ABR-217620 demonstrates selective interaction with TRBV7-9. Binding of TRBV7-9 and TRBV6-5 to ABR-217620. Samples were injected (2 min at 20 µL/min) over ABR-217620 (density ~724 RU) in the concentration range 0.0625-1 µM. The surface was regenerated by dissociation in running buffer. Only TRBV7-9 showed detectable binding to ABR-217620. (TIF) [file pone.0079082.s011.tif]
